# Vapor Deposition Strategies for Copper‐Based Electrocatalysts in CO_2_ Reduction Applications

**DOI:** 10.1002/cssc.202500813

**Published:** 2025-09-29

**Authors:** Lovelle Rhoy Manpatilan, Stefano Bianco, Giancarlo Cicero, Juqin Zeng, Elena Maria Tresso

**Affiliations:** ^1^ Dipartimento di Scienza Applicata e Tecnologia Politecnico di Torino Torino 10129 Italy; ^2^ Center for Sustainable Future Technologies Istituto Italiano di Tecnologia Torino 10144 Italy

**Keywords:** CO_2_ electrochemical reduction, CO2RR, copper electrocatalysts, copper films, vapor deposition

## Abstract

The advent of copper‐based electrocatalysts has significantly advanced the electrochemical conversion of CO_2_ into valuable multicarbon fuels and chemicals. Among various synthesis methods, vapor deposition techniques offer a facile and innovative approach to produce catalysts with high conformance and precise control of morphology, thickness, and composition. This article provides a review of the application of the main vapor deposition techniques for the development of copper‐based catalysts and electrodes for tuned CO_2_ reduction reaction (CO2RR). The first part introduces the CO2RR principles, electrolyzer types and components, and Cu electrocatalysts, highlighting their critical role in efficient CO2RR. Then, the principles of physical vapor deposition (PVD), chemical vapor deposition (CVD), and atomic layer deposition (ALD) are introduced, including the process variations and comparative advantages. This review highlights the most promising results obtained with Cu‐based catalysts and electrodes for CO2RR via vapor deposition, with a particular focus on monometallic, bimetallic, single‐atom, and modified Cu catalysts. The review concludes with our perspectives on applying vapor deposition techniques for advanced catalyst preparation. We emphasize that combining these techniques offers unique opportunities to fine tune the material properties at the nanoscale, thereby optimizing active sites for improved CO_2_ conversion selectivity and activity.

## Graphical abstract

This review explores the innovative application of vapor deposition techniques such as physical vapor deposition, chemical vapor deposition, and atomic layer deposition for fabricating copper‐based electrocatalysts for CO_2_ electrochemical reduction.

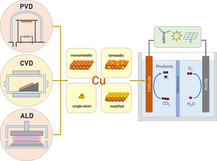

## 1 Introduction

The rapid increase of anthropogenic greenhouse gases (GHGs) in the atmosphere has been attributed to shifts in climate patterns and record‐breaking worldwide temperatures.^[^ ^1^, ^2^ ^]^ In 2024, the global annual average temperature reached 1.55 ± 0.13 °C more than preindustrial levels (1850–1900), which has approached the 1.5 °C limit set by the Paris Agreement.^[^ ^3^ ^]^ Carbon dioxide (CO_2_) comprises over 70% of the emitted GHGs, while methane, nitrous oxide, and fluorinated gases are the other major contributors.^[^ ^4^ ^]^ Consequently, a number of initiatives have been implemented to mitigate CO_2_ emissions and close the carbon cycle, including carbon capture and utilization (CCU) technologies.^[^ ^5^, ^6^ ^]^ These encompass electrochemical, thermal, photocatalytic, and biochemical approaches to convert CO_2_ into value‐added products, like chemicals, synthetic fuels, building materials, and polymers.^[^ ^7^, ^8^ ^]^ However, CO_2_ is an inert and extremely stable molecule, thus the need for energy‐expensive methods for its conversion.^[^ ^9^ ^]^

Electrochemical reduction is one of the promising and sustainable methods of CO_2_ conversion, as it utilizes renewable energy and inexpensive aqueous electrolytes to generate valuable products at ambient temperature and pressure. These products can be classified into single‐carbon (C_1_) and multicarbon (C_2+_) compounds which comprise of carbon monoxide (CO), formic acid (HCOOH), methane (CH_4_), methanol (CH_3_OH), acetic acid (CH_3_COOH), ethylene (C_2_H_4_), and ethanol (C_2_H_5_OH).^[^ ^10^, ^11^, ^12^ ^]^ The main bottlenecks of electrochemical CO_2_ reduction are slow reaction kinetics, due to the high energy barriers of the reaction, and poor selectivity, resulting from undesired side reactions.^[^ ^13^ ^]^ Thus, the electrocatalyst plays a key role in optimizing the efficiency and selectivity of the CO_2_ reduction process.^[^ ^14^, ^15^ ^]^

Among the various types of metallic electrocatalysts, copper (Cu) has been identified as the only metal to generate C_2+_ products from CO_2_ reduction, with active sites that catalyze the formation of up to 16 different products. Achieving high activity and selectivity with Cu‐based electrocatalysts depends heavily on their structure, composition, and surface properties.^[^ ^11^, ^16^, ^17^ ^]^ Fabrication of Cu‐based catalysts has mainly relied on conventional wet chemical, electrochemical, and thermal methods. Vapor deposition techniques, which encompass physical vapor deposition (PVD), chemical vapor deposition (CVD), and atomic layer deposition (ALD), have emerged as alternative methods for synthesizing electrocatalysts. These methods allow for the fabrication of a wide range of Cu catalyst architectures, such as monometallic films with controlled morphology and thickness, bimetallic catalysts with tuned composition and homogeneity, single‐atom catalysts, and chemically or surface‐modified Cu catalysts. By enabling tailored catalyst designs, vapor deposition methods offer new pathways to optimize active sites and improve CO_2_ reduction performance. This review explores the application of vapor deposition techniques of Cu‐based electrocatalysts for CO_2_RR, highlighting recent advances and key challenges.

## 2 Electrochemical CO_2_ Reduction

This section covers the basic principles behind CO2RR, including the key reactions that occur at the cathode, as well as the challenges related to overpotentials and product selectivity. It also introduces the metrics used to evaluate CO2RR performance and provides an overview of the various reactor types employed in CO_2_ reduction systems, such as the H‐cell, flow cell, and membrane electrode assembly.

### 2.1 Electrochemical CO_2_ Reduction Principles

The electrochemical reduction of CO_2_ utilizes a cathode and an anode, immersed in electrolyte and separated by an ion‐exchange membrane (IEM), and connected by an external power source. At the anode, the oxygen evolution reaction (OER) occurs by oxidizing water, generating electrons that are supplied to the cathode via an external circuit. At the cathode, where the electrocatalyst is contained, the supplied electrons interact with protons from the electrolyte to facilitate the CO_2_ reduction reactions.^[^ ^18^ ^]^ The CO2RR in aqueous solutions involves complex reduction pathways, with multielectron and proton transfer steps to generate various products.^[^ ^19^, ^20^ ^]^ In addition, the competing HER occurs at the cathode by reducing water.^[^ ^21^ ^]^ **Table** **1** summarizes the main possible reactions occurring at the cathode of a typical CO_2_ electrolyzer, along with the respective reaction potential versus the reversible hydrogen electrode (RHE) at 1.0 atm and 25 °C.

**Table 1:** Standard electrochemical potentials of reactions occurring at the cathode in CO_2_ electrolyzers.^[^ ^6^, ^20^, ^22^ ^]^

Product	Reaction	E^0^ [V versus RHE]
Formic acid	CO _2_ + 2 H ^+^ + 2e ^−^ → HCOOH _(aq)_	−0.250
Carbon monoxide	CO_2_ + 2H^+^ + 2e^−^ → CO_(g)_ + H_2_O	−0.106
Methanol	CO_2_ + 6H^+^ + 6e^−^ → CH_3_OH_(aq)_ + 2H_2_O	0.016
Methane	CO_2_ + 8H^+^ + 8e^−^ → CH_4(g)_ + 2H_2_O	0.169
Acetic acid	2CO_2_ + 8H^+^ + 8e^−^ → CH_3_COOH_(aq)_ + 2H_2_O	0.114
Ethylene	2CO_2_ + 12H^+^ + 12e^−^ → C_2_H_4(g)_ + 4H_2_O	0.064
Ethanol	2CO_2_ + 12H^+^ + 12e^−^ → C_2_H_5_OH_(aq)_ + 3H_2_O	0.084
Hydrogen	2H^+^ + 2e^−^ → H_2(g)_	0.00

Although the standard potentials for CO2RR are comparable to those of the hydrogen evolution reaction (HER), significantly higher overpotentials are typically needed to achieve reasonable reaction rates for CO2RR. Furthermore, since the standard potentials for all CO2RR products are similar, achieving high selectivity toward a specific product remains a significant challenge.^[^ ^22^, ^23^ ^]^ To overcome the overpotential and selectivity issues, particularly for C_2+_ products, apart from improving the intrinsic catalytic properties of Cu, external factors like electrode surface characteristics and the reaction conditions also call for much attention since they remarkably influence CO2RR. These conditions encompass the applied potential, electrolyte composition, local pH levels, and local CO_2_ concentration.^[^ ^24^, ^25^ ^]^

To assess the performance of a CO_2_ reduction process, key metrics such as product selectivity, electrode activity, and stability are established. Product selectivity is measured by faradaic efficiency (FE) which refers to the fraction of the electrical current contributing to the formation of a specific product during steady‐state electrolysis. Electrode activity is quantified by the partial current density at a specific potential, which is calculated by multiplying the total current density by the FE of the specific product. It is also quantified by the overpotential at a specific current density, defined as the absolute difference between the actual potential and the standard potential to produce the product. Stability is evaluated by the duration over which a CO2RR system can sustain a stable current density and FE for the target product.^[^ ^26^ ^]^ Quantification of products is essential for defining the FE and partial current density of each product. The most common detection method for gaseous products is gas chromatography (GC), while nuclear magnetic resonance spectroscopy (NMR) and high‐performance liquid chromatography (HPLC) are widely used for liquid phase product detection.^[^ ^27^ ^]^

### 2.2 Electrochemical CO_2_ Reduction Reactor Types

**Figure 1:**
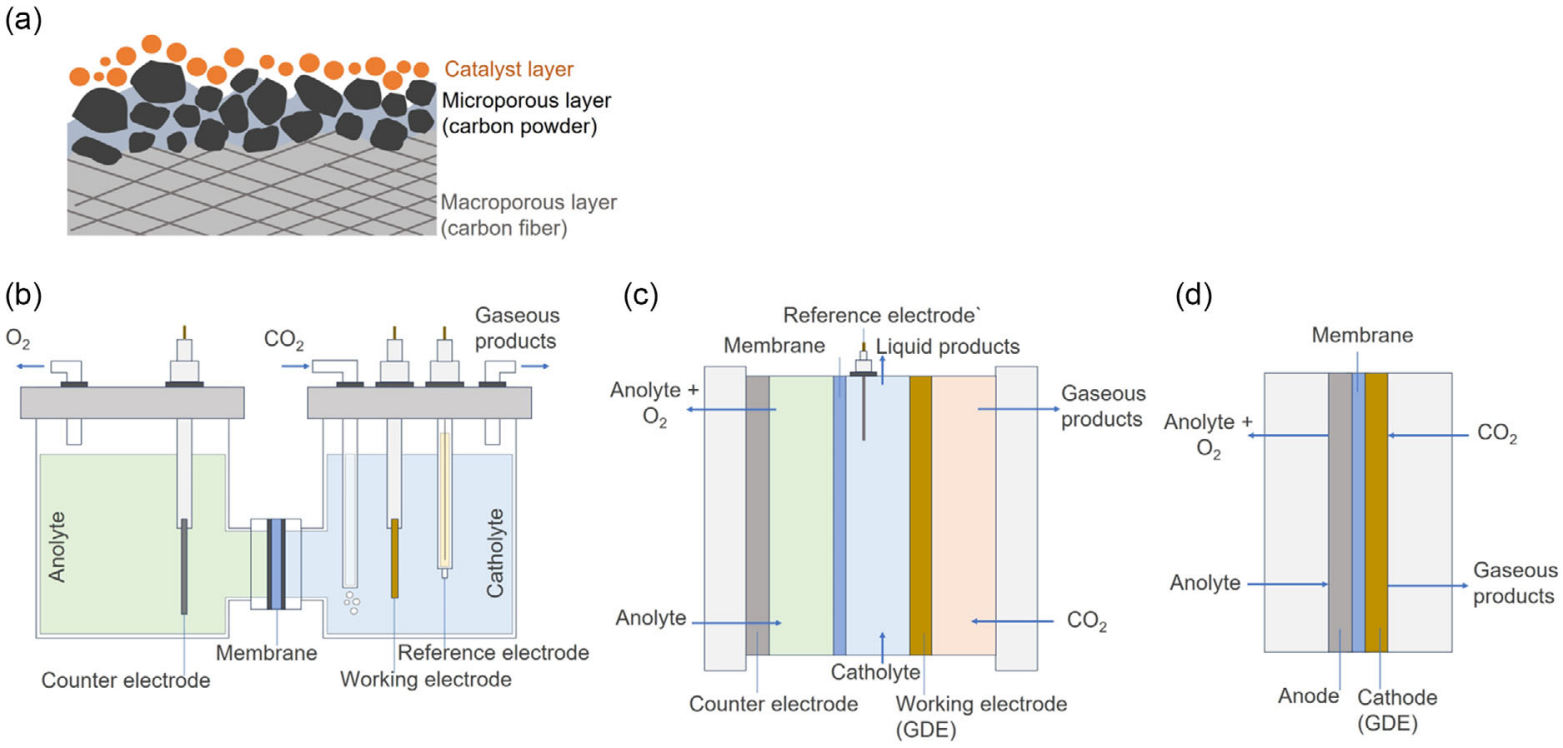
Schematic of a) gas diffusion layer with a catalyst layer to form a gas diffusion electrode, b) H‐type cell, c) flow cell, and d) MEA cell.

Several reactor designs are available for electrochemical CO_2_ reduction, each facilitating CO2RR at the cathode and OER at the anode.^[^ ^28^ ^]^ The cathode is commonly comprised of a substrate and a catalyst loaded on it (**Figure** **1**a). The reactor types mainly include H‐type cells, flow cells, and membrane electrode assemblies (Figure 1b–d).^[^ ^29^ ^]^

#### 2.2.1 H‐Cell

The H‐type cell, or H‐cell, is the simplest and least expensive electrolyzer commonly used for fundamental CO2RR measurements (Figure 1b).^[^ ^30^ ^]^ It is comprised of anodic and cathodic compartments, separated by an IEM. In a three‐electrode setup, the anodic compartment contains the counter electrode, while the cathodic compartment contains the reference electrode and the working electrode with the electrocatalyst. Prior to electrochemical reduction, CO_2_ is bubbled and saturated into the aqueous electrolyte and transferred to the cathodic compartment. However, due to the low solubility of CO_2_ in aqueous electrolytes (≈34 mM under ambient conditions), the current densities obtained from this system are typically less than − 100 mA cm^−^^2^.^[^ ^29^, ^31^ ^]^ In addition, H‐cells experience high ohmic losses due to the large distance between the cathode and anode.^[^ ^28^ ^]^ Neutral electrolytes, typically KHCO_3_, are commonly used, further increasing the overpotentials due to their relatively low ionic conductivity.^[^ ^30^ ^]^

#### 2.2.2 Flow Cell

A flow cell (Figure 1c) is a reactor type that allows the continuous flow of reactants and products, effectively overcoming the mass transport limitations that are inherent to an H‐cell. For CO_2_ reduction applications, flow cells typically include a dedicated chamber for supplying gaseous CO_2_ to the gas diffusion electrode (GDE) and collecting gaseous products.^[^ ^32^ ^]^ These cells are generally categorized as either membrane‐based flow cells or microfluidic flow cells. Membrane‐based flow cells, similar to H‐cells, use an IEM to separate the anolyte and catholyte, allowing independent flow in each compartment. This configuration is promising for large‐scale commercial applications due to its compact structure, high current densities, and efficient CO2RR performance.^[^ ^33^, ^34^ ^]^ Microfluidic flow cells, on the other hand, utilize an ultrathin electrolyte channel (<1 mm thick) to separate the electrodes without requiring membranes. The thin channels provide laminar flow conditions, forming a liquid‐liquid interface that acts like a virtual membrane for the effective separation of the products and reactants.^[^ ^33^, ^35^ ^]^ This design offers better control over the composition and pH of the electrolyte, while also minimizing common issues like cathode flooding, anode dry‐out, and membrane handling.^[^ ^36^, ^37^ ^]^

To achieve higher CO2RR current density values, the GDE has been utilized in flow cells and zero‐gap electrolyzers. Mahmood et al.^[^ ^38^ ^]^ pioneered the use of GDEs for the electrochemical reduction of CO_2_ in 1987, achieving current densities exceeding − 100 mA cm^−^^2^. A conventional GDE is composed of a catalyst layer (CL) coated onto a carbon‐based gas diffusion layer (GDL). The GDL itself consists of a microporous layer comprising carbon powder and a macroporous layer containing carbon fibers (Figure 1a).^[^ ^39^ ^]^ This porous structure allows a three‐phrase reaction interface between CO_2_ gas, liquid electrolyte, and catalyst, which is commonly considered essential for CO_2_ electrochemical reactions, although two‐phase mechanisms have also been proposed. During CO2RR, the catalyst layer of the GDE is in direct contact with the electrolyte, while the porous layer facilitates CO_2_ gas access. This design offers a shorter diffusion pathway for CO_2_ to reach the catalyst surface.^[^ ^30^, ^40^, ^41^ ^]^ The GDL also provides mechanical support for the CL and ensures electronic conductivity by facilitating electron flow from the current collector and external circuit to the CL.^[^ ^42^, ^43^ ^]^ A commonly encountered issue in GDE operation is electrolyte flooding, where the electrolyte penetrates the electrode structure. This obstructs CO_2_ diffusion pathways in the CL, reducing current density and promoting the competing HER.^[^ ^44^, ^45^ ^]^ To mitigate flooding, most commercial GDLs are treated with polytetrafluoroethylene (PTFE) to increase hydrophobicity, with a tradeoff of lower conductivity and CO_2_ transport.^[^ ^46^ ^]^

#### 2.2.3 Membrane Electrode Assembly

The membrane electrode assembly (MEA), widely used for proton exchange membrane fuel cells and water electrolyzers, is another type of reactor that utilizes GDE.^[^ ^47^, ^48^ ^]^ The MEA cell, also called a zero‐gap cell (Figure 1d), operates similarly to a flow cell but differs in that the catholyte chamber is removed.^[^ ^31^, ^49^ ^]^ In this zero‐gap setup, only the CO_2_ gas and anolyte streams are present, with the membrane placed directly between the cathode and anode, facilitating the three‐phase reaction interface for the relevant reactions to occur.^[^ ^22^ ^]^ This design addresses many of the flow cell‐related issues, particularly electrolyte ohmic losses, electrolyte consumption due to its neutralization with CO_2_, catalyst fouling from electrolyte impurities, and electrolyte flooding.^[^ ^45^, ^50^ ^]^ As a result, the MEA offers improved stability in cell voltage and product selectivity, making it well‐suited for industrial applications.^[^ ^50^ ^]^

### 2.3 Electrochemical CO_2_ Reduction Reactor Components

#### 2.3.1 Membranes

The IEM used in electrochemical cells prevents electrolyte cross‐contamination and electrode short‐circuit, while maintaining ionic conductivity by allowing ion transport between the compartments. These membranes are generally classified as cation exchange membranes (CEMs), which contain fixed anions to allow counter‐cation mobility, and anion exchange membranes (AEMs), which contain fixed cations to allow counter‐anion mobility.^[^ ^28^, ^51^, ^52^ ^]^ Conventional CEMs offer high ionic conductivity through proton transport but suffer from excessive swelling and low FE due to increased HER caused by lowered local pH at the cathode. In contrast, AEMs allow OH^−^ transport at basic environments, promoting higher faradaic efficiencies by suppressing HER. However, the major drawbacks of AEMs include high CO_2_ loss due to carbonate/bicarbonate crossover and salt precipitation due to high local pH, and liquid products crossover to anodic side. To address these limitations, bipolar membranes (BPMs) have been developed by combining a cation exchange layer (CEL) with an anion exchange layer (AEL). This design enables the formation of distinct pH environments at the cathode and anode, while minimizing ion crossover. In forward bias mode (CEL at the anode), cations and anions migrate through their domains and combine at the interface to form water, supporting stable operation but requiring management of gas evolution. In reverse bias mode (CEL at the cathode), an electric field drives water dissociation at the interface, limiting carbonate crossover and allowing liquid CO_2_ feedstocks, though the acidic cathode increases the risk of HER.^[^ ^28^, ^53^, ^54^ ^]^

#### 2.3.2 Electrolytes

The pH of the electrolyte also impacts CO2RR performance by influencing reactant availability, competing side reactions like HER, catalyst stability, and salt deposition at the cathode. Using highly alkaline electrolytes like KOH can advance the formation of hydrophilic carbonate and bicarbonates as CO_2_ reacts with OH^−^, causing electrode flooding, salt deposition on the backside of cathode, and decreased energy efficiencies.^[^ ^44^, ^55^ ^]^ Neutral electrolytes such as KHCO_3_ lessen the formation of carbonate precipitates, while having decreased HER compared to acidic electrolytes.^[^ ^56^, ^57^ ^]^ However, KHCO_3_ shows lower ionic conductivity and FE for C_2_ products, largely compromising the energy efficiency of the process. Meanwhile, acidic electrolytes eliminate salt accumulation and flooding and offer lower electrolyte resistance, while they strongly favor HER due to the enrichment of protons near the active sites.^[^ ^57^ ^]^ The careful selection of membranes and electrolytes is of great importance for the optimization of CO2RR performance, as they collectively influence ionic conductivity, faradaic efficiency, and the balance of competing reactions.^[^ ^58^ ^]^

#### 2.3.3 Anodes

The anode contains electrocatalysts, typically based on Ir, Ni, or Pt, that drive the OER during CO_2_ electrolysis. The choice of anode catalyst must account for not just OER activity but also chemical stability in dynamic environments, such as the gradual neutralization of the alkaline electrolyte during continuous operation. Although OER is widely used due to its simplicity and well‐understood nature, it requires a high thermodynamic potential (1.23 V versus RHE), incurs additional overpotential due to sluggish kinetics, and generates low‐value oxygen. As a result, increasing interest is being directed toward alternative anodic reactions, like alcohol oxidation (e.g., glycerol to formate), which occur at lower potentials and yield valuable coproducts, thereby improving the energy efficiency and economic viability of CO2RR systems.^[^ ^59^ ^]^

#### 2.3.4 Copper‐Based Cathodes

The unique ability of copper to convert CO_2_ into useful hydrocarbons and alcohols is primarily due to its favorable binding energy to *CO, a key intermediate for further reduction into multi‐carbon products.^[^ ^22^ ^]^ According to Bagger et al.,^[^ ^60^ ^]^ Cu is the sole metal with a negative adsorption energy for *CO but a positive adsorption energy for *H. This allows Cu to effectively adsorb and stabilize *CO, while suppressing the competing HER. In comparison, metals like mercury (Hg), silver (Ag), gold (Au), palladium (Pd), and zinc (Zn) promote CO formation, while tin (Sn), lead (Pb), bismuth (Bi), and indium (In) favor HCOOH formation over CO. Thus, Cu stands out for its ability to facilitate reduction processes beyond two‐electron products, unlike these metals that predominantly yield CO or HCOOH as terminal products.^[^ ^15^, ^61^ ^]^

Extensive research has been dedicated to modifying Cu‐based electrocatalysts to enhance their CO_2_ reduction performance, as highlighted in recent comprehensive reviews.^[^ ^22^, ^24^, ^26^, ^31^, ^62^, ^63^, ^64^, ^65^, ^66^ ^]^ Such modification strategies feature controlling the particle size and shape,^[^ ^67^ ^]^ oxidation states,^[^ ^68^ ^]^ exposed facets,^[^ ^69^, ^70^ ^]^ coordination number,^[^ ^71^ ^]^ grain boundaries,^[^ ^72^ ^]^ surface area,^[^ ^73^ ^]^ and composition.^[^ ^74^, ^75^ ^]^ As a result, a wide array of Cu‐based catalysts with diverse structures has been developed, namely oxides,^[^ ^68^ ^]^ single‐atom catalysts,^[^ ^76^ ^]^ functionalized catalysts,^[^ ^77^ ^]^ doped catalysts,^[^ ^78^ ^]^ and bimetallic catalysts.^[^ ^31^, ^63^, ^64^, ^65^ ^]^ Among these, bimetallic catalysts formed by introducing other metals to Cu have attracted considerable interest due to their distinct properties compared to monometallic Cu. These differences in property are attributed to electronic structure modifications, which introduce new active sites that optimize the binding strength of reaction intermediates, enabling control over product selectivity.^[^ ^64^, ^79^ ^]^ Moreover, the crystal facets of Cu itself play a significant role in product selectivity. The Cu(100) facet is thermodynamically active for C—C coupling, promoting the formation of C_2+_ products, whereas Cu(111) favors C_1_ product formation.^[^ ^66^, ^69^, ^70^ ^]^

In terms of synthesis of Cu‐based electrocatalysts, the most widely used methods include electrochemical,^[^ ^77^, ^80^ ^]^ wet chemical,^[^ ^81^, ^82^ ^]^ solvothermal,^[^ ^74^, ^75^ ^]^ and thermal treatment routes,^[^ ^83^, ^84^ ^]^ each offering unique advantages in terms of control over morphology and composition. These methods are often favored for their simplicity, relatively low cost, and accessibility of precursors and equipment.^[^ ^85^, ^86^ ^]^ However, as these techniques typically employ liquid media, additional processing steps like separation, purification, or drying are required prior to their utilization as electrocatalysts.^[^ ^87^ ^]^ Vapor deposition techniques, which are discussed further in the following sections, are promising methods for the synthesis of Cu‐based catalysts. Despite the need for advanced equipment and specialized precursors, these techniques enable the facile and direct deposition of active materials onto substrates.^[^ ^88^, ^89^ ^]^ They also allow for the even coating of materials with high surface area and intricate nanostructures, making them highly suitable for a broad range of applications, especially in semiconductor manufacturing, nanofabrication, and even catalysis.^[^ ^89^ ^]^

## 3 Vapor Deposition Techniques

### 3.1 Physical Vapor Deposition

PVD is an atomistic deposition process in which a solid or liquid source is vaporized, transported through a vacuum or low‐pressure gaseous environment, and condensed onto a substrate to form a film.^[^ ^90^ ^]^ The two most commonly used PVD techniques are evaporation and sputtering.^[^ ^91^ ^]^ These are considered line‐of‐sight techniques due to their limited ability in coating substrates with high surface areas or high aspect ratios. Pulsed laser deposition (PLD) is another type of PVD that offers improved control over film growth. A summary of several PVD techniques for the fabrication of Cu films is provided in **Table** **2**.

**Table 2:** Examples of PVD of copper films.

Process	Process conditions	Pressure [mbar]a)	Growth rate [nm min^−1^]b)	Substrate	Ref.
Thermal evaporation	950–1300 °C source	≈6.6 × 10^−6^	0.6–180	–	[95]
Electron beam evaporation	Room temp. source, 80 mA beam current, 8 kV	3	–	TiO_2_/glass	[181]
DC magnetron sputtering	135 W power	0.0075	12	Glass	[97]
DC magnetron sputtering	1000 W power	0.003	417	Glass/Si	[96]
High‐power impulse MS	40 μs pulse at 120 Hz, 1000 W ave. power	0.003	48	Glass/Si	[96]
Burst regime MS	0.125 Hz pulses with 1s pulse train, 1000 W ave. power	0.003	150	Glass/Si	[96]
RF sputtering	40 W power	<0.001	6	Al_2_O_3_	[98]
PLD	180 °C substrate, 20 ns pulses at 10 Hz	3 × 10^−7^	0.5	MgO	[182]

a) Base pressure values are converted to mbar unit;

b) Growth rate values are converted to nm per minute unit.

#### 3.1.1 Evaporation

**Figure 2:**
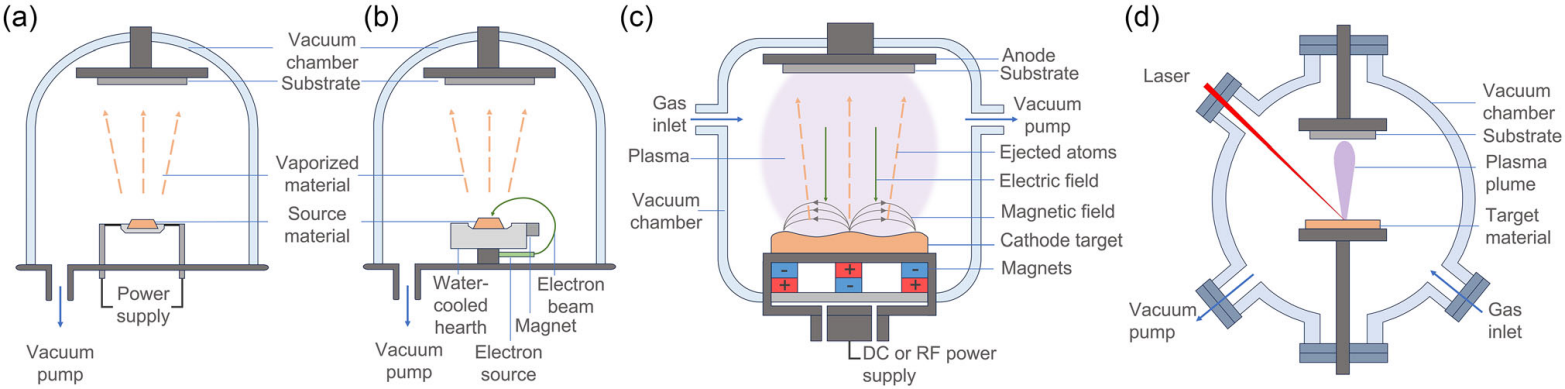
Schematic of a) thermal evaporation, b) electron beam evaporation, c) magnetron sputtering, and d) pulsed laser deposition.

Evaporation is a thermal deposition technique where the material source is heated until it vaporizes, and the vapor is transported under high vacuum to form a thin film on a substrate.^[^ ^89^ ^]^ Evaporation can be classified based on the method used to heat the evaporant material: thermal evaporation and electron beam (EB) evaporation. Thermal evaporation (**Figure** **2**a) involves passing a large current through a resistive wire, crucible, or boat to heat and vaporize the source material. In comparison, EB evaporation (Figure 2b) uses a high‐energy electron beam to bombard and evaporate the source material.^[^ ^92^, ^93^, ^94^ ^]^

Evaporation PVD requires a high temperature for the melting of copper at 1083 °C and high vacuum levels (≈10^−^^6^ mbar base pressure). For this technique, the growth rate increases with the evaporation temperature of the Cu source.^[^ ^95^ ^]^ The typical sources for the PVD of Cu films are commercial Cu wires and cathodes with a very high purity of 99.95–99.997%.^[^ ^95^, ^96^, ^97^, ^98^ ^]^ However, custom targets for the PVD of Cu‐based films can be created through pressing and annealing Cu‐based powders.^[^ ^99^, ^100^ ^]^

#### 3.1.2 Sputtering

Sputter deposition, or sputtering, is a nonthermal process in which surface atoms are ejected from a target by momentum transfer of energetic bombarding particles, typically ions from a plasma.^[^ ^90^, ^101^ ^]^ This phenomenon was first recorded by W. R. Grove in 1852, when an iron oxide deposit was formed on a silver substrate from a steel needle cathode in a vacuum chamber filled with an air‐hydrogen mixture, and reversing the electrode polarity removed the deposit.^[^ ^102^, ^103^, ^104^ ^]^ Among the widely used types of sputtering techniques are direct current (DC), radio frequency (RF), and magnetron sputtering (MS). Direct current sputtering uses a DC‐powered diode and is primarily used to deposit electrically conductive materials. For RF sputtering, an alternating current (AC) power source is used at high frequency to enable deposition also on semiconductors and insulators.^[^ ^105^, ^106^ ^]^ Magnetron sputtering (Figure 2c), which typically employs either DC or RF power sources, utilizes a magnetic field to confine electrons near the target material and enhance the ionization of the sputtering gas. This allows a higher deposition rate, lower discharge voltage, and expanded operating pressure range versus diode‐based sputtering.^[^ ^106^, ^107^ ^]^

Additional sputtering modes, such as reactive sputtering and cosputtering, can be used to deposit compound films. Reactive sputtering employs reactive gases to form nitrides, oxides, carbides, etc., of the sputtered material, with the gas flow rate influencing the stoichiometry of the final product.^[^ ^108^, ^109^ ^]^ Cosputtering uses multiple targets to simultaneously deposit alloys or doped films, with the sputtering power for each target adjustable to achieve the desired stoichiometry.^[^ ^110^ ^]^ Multilayered films can also be fabricated through the alternate sputtering of different targets onto the same substrate.^[^ ^111^ ^]^ Ion‐beam‐assisted deposition (IBAD), which utilizes an angled ion beam source, can also be used to induce substrate cleaning, preferential damage, and preferential sputtering for strongly textured films.^[^ ^112^ ^]^

The growth rate for sputtering can be controlled by adjusting the applied discharge power. Moreover, various discharge power regimes for magnetron sputtering, such as continuous direct current, high power impulse (HIPIMS), and burst regimes, can result in films with different morphologies and characteristics. Solovyev et al.^[^ ^96^ ^]^ compared these regimes for Cu film deposition under equal average power. HIPIMS produced the highest plasma density and yielded denser, smoother, less columnar films than DCMS, but at the cost of a lower deposition rate due to strong ion back‐attraction. In contrast, the burst mode, employing low‐frequency pulsed discharges with alternating polarity, enabled improved plasma modulation and exhibited dense, homogeneous films without columnar grains and with the lowest electrical resistivity.

#### 3.1.3 Pulsed Laser Deposition

In PLD, a pulsed laser is used to ablate a target surface, creating a plasma plume of ejected species. These species undergo gas phase reactions with the surrounding atmosphere before forming a thin film on the substrate (Figure 2d).^[^ ^113^ ^]^ PLD is a versatile technique, offering control of substrate‐to‐target distance and operating pressure, which can range from ultrahigh vacuum to the mbar level. This enables precise control of the film density and high suitability for materials that have complex compositions. However, PLD is susceptible to the formation of large clusters during laser ablation, potentially causing inhomogeneity and surface roughness in the deposited film.^[^ ^114^ ^]^

### 3.2 Chemical Vapor Deposition

**Figure 3:**
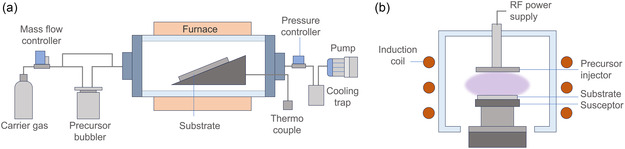
Schematic of a) horizontal hot‐wall MOCVD setup and b) vertical cold‐wall plasma‐enhanced MOCVD reactor.

CVD involves the chemical reaction of gaseous precursors within a reaction chamber, leading to the formation of a target film on the substrate surface. The chamber is activated by heat, light, or plasma to enable the chemical reactions or precursor dissociation for film formation.^[^ ^115^, ^116^ ^]^ As a chemically driven process, CVD allows adjustable deposition rates and produces high‐quality products with excellent substrate conformity.^[^ ^117^ ^]^ CVD can be classified from the following categories: reactor type (horizontal or vertical), energy source (thermally activated (**Figure** **3**a), plasma‐enhanced (Figure 3b), photo‐assisted, or laser‐assisted), working pressure (low‐pressure or atmospheric pressure), heating methods (hot‐wall or cold‐wall), and precursors (metal–organic or aerosol‐assisted).^[^ ^115^, ^117^, ^118^ ^]^ Inert gases like N_2_, Ar, or He are often used as carrier gases to transport the precursors toward the reaction chamber, while reactive gases like H_2_, O_2_, and NH_3_ can facilitate oxidation or reduction reactions.^[^ ^119^ ^]^

The underlying mechanisms governing Cu CVD involve complex thermal decomposition pathways, typically governed by disproportionation reactions or reduction processes with a reductant such as H_2_ gas.^[^ ^120^ ^]^ While CVD is traditionally characterized by the reaction of a volatile precursor with a secondary gas at the substrate surface to yield the desired film, certain copper precursors can bypass this requirement. Due to the inherent ease with which copper compounds are reduced to the metallic state, many of these precursors function as single‐source systems, wherein the ligand framework itself serves as the internal reducing agent. In the early stages of CVD, Cu typically forms as discrete islands that can later coalesce into a continuous film with increased deposition time or precursor flow rate.^[^ ^116^, ^121^, ^122^ ^]^ The deposition temperature for CVD processes plays a critical role in determining the growth rate and composition of Cu films. While higher temperatures typically increase growth rates and particle sizes, they can also lead to a higher degree of contamination.^[^ ^123^, ^124^ ^]^ Morphology is also temperature dependent; lower temperatures tend to produce smooth films, while higher temperatures can produce porous and columnar grown particles.^[^ ^125^ ^]^

#### 3.2.1 Metal–Organic CVD

Metal–organic CVD (MOCVD) is a subset of CVD that utilizes volatile metal–organic compounds to deposit high‐quality films at lower temperatures.^[^ ^116^ ^]^ While Cu halide precursors like CuCl require a high vaporization temperature of around 300–350 °C, metal–organic precursors can have vaporization temperatures below 100 °C, depending on the polarity of the organic ligands.^[^ ^124^, ^126^ ^]^ The commonly used Cu metal–organic precursors occur as Cu(I) or Cu(II) compounds with different ligands such as β‐diketonates [Cu(acac)_2_ and Cu(hfac)_2_],^[^ ^127^, ^128^ ^]^ aminoalkoxides [Cu(dmap)_2_ and Cu(deap)_2_],^[^ ^129^ ^]^ amidinates [Cu(^i^Pr‐Me‐amd)]_2_,^[^ ^123^ ^]^ and formate [Cu(COOH)_2_],^[^ ^120^, ^130^ ^]^ and Cu(I) precursors, particularly amidinates, offer higher volatility and sufficient reactivity for low‐temperature Cu film deposition. Cu(II) precursors, despite their ease of synthesis and good thermal stability, are limited by low vapor pressure, reduced reactivity, and the risk of carbon, oxygen, nitrogen, or fluorine contamination due to incomplete reduction for the case of diketonates.^[^ ^131^ ^]^ Most of these precursors are solid at ambient conditions and require elevated temperatures to vaporize, which can pose challenges for process repeatability, reliability, and growth rates. As a result, liquid precursors, particularly Cu(hfac)(tmvs), are often used in commercial applications as they offer better film qualities with higher growth rates and lower deposition temperatures.^[^ ^132^, ^133^, ^134^ ^]^

#### 3.2.2 Other CVD Processes

Plasma‐enhanced CVD (PECVD) allows the deposition of smooth, low electrical resistivity Cu films at lower deposition temperatures, as compared to the rough, high resistivity films typically produced by conventional CVD. The enhanced smoothness is attributed to the plasma‐generated reactive species that excite or dissociate the Cu precursor, leading to a higher Cu nucleation density. In contrast, thermal CVD suffers from low nucleation probability that results in the growth of separated islands.^[^ ^135^ ^]^ Laser‐assisted CVD enables localized copper deposition using focused laser beams to induce localized decomposition of Cu precursors, which is particularly useful for selective area deposition.^[^ ^136^ ^]^ Photo‐assisted CVD employs UV light to assist in the decomposition of the precursors at lower temperatures than thermal CVD.^[^ ^137^ ^]^ Aerosol‐assisted CVD allows the use of nonvolatile precursors through the use of a nebulizer to generate aerosol droplets for simplified and cost‐effective precursor delivery to the reactor via pressurized carrier gas.^[^ ^138^ ^]^

Hot wire CVD is a technique where a heated filament, such as tungsten, thermally activates the precursors in the gas phase before they reach the substrate, enabling a higher growth rate than conventional MOCVD and deposition on insulating surfaces. Direct liquid injection (DLI) CVD complements these methods by precisely injecting a liquid precursor stored at room temperature into a heated zone within the reactor for vaporization and controlled delivery, allowing uniform deposition, unlike traditional bubbler‐based systems which rely on precursor vapor pressure and carrier gas bubbling for vapor transport.^[^ ^133^ ^]^ Pulsed CVD introduces the precursor and reactant sequentially in pulses to allow controlled film growth and improved film quality, at a faster deposition rate compared to ALD.^[^ ^131^ ^]^ In terms of mode of heating, hot‐wall CVD offers uniform temperature control by heating the entire chamber but suffers from wall deposition and reactant depletion, whereas cold‐wall CVD heats only the substrate, minimizing wall deposition, though reduced‐pressure operation is often required to suppress thermal convection and achieve uniform coatings.^[^ ^115^ ^]^

Comprehensive reviews of Cu CVD precursors and processes are provided by Gordon et al.,^[^ ^116^ ^]^ Rickerby and Steinke,^[^ ^124^ ^]^ and Vertoprakhov and Krupode.^[^ ^139^ ^]^ Several examples of CVD techniques for the fabrication of Cu films are shown in **Table** **3**.

**Table 3:** Examples of CVD of copper films.

Process	Cu precursor	Precursor temp [°C]	Deposition temp [°C]	Carrier/reactant gas	Pressure [mbar]a)	Growth rate [nm min^−1^]b)	Substrate	Ref.
Hot‐wall CVD	CuCl	300–350	350–500	H_2_/Ar	13	5–8	Si/SiO_2_	[126]
MOCVD	Cu(acac)_2_	220	220–400	H_2_	1013	6–20	Glass	[127]
Cold‐wall MOCVD	Cu(hfac)_2_	65–85	310–390	H_2_/Ar	2.7–13.3	20–60	SiO_2_	[128]
Cold‐wall MOCVD	Cu(tbaoac)_2_	90–150	225–320	Ar	13.3	2.7	SiO_2_	[183]
Cold‐wall MOCVD	Cu(hfac)_2_.TMEDA	70	250–550	H_2_O/O_2_	10	–	Si	[184]
Laser‐assisted CVD	Cu(hfac)_2_	100	130	H_2_/Ar	1013	20–120	Si/SiO_2_	[136]
Photo‐assisted CVD	Cu(thd)_2_	140	150–390	He	13.3		Si,Si_3_N_4_, Al	[137]
Thermal and PECVD	CuCOOH	115–125	220–320	Ar	1.5	0.3	Si/SiO_2_	[185]
MOCVD	Cu(COOH)_2_	140–180	300–350	H_2_/Ar	20	–	Si/TiN	[120]
MOCVD	Cu(hfac)(tmvs)	40	200–250	H_2_/N_2_	0.7	70–80	Si	[134]
Hot Wire, DLI MOCVD	Cu(hfac)(tmvs)	Room temp	180–220	N_2_	–	≈6–15	Si/TiN/W	[134]

a) Deposition pressure values are converted to mbar unit;

b) Growth rate values are converted to nm per minute unit.

### 3.3 Atomic Layer Deposition

ALD is a specific type of CVD, whereby the continuous flow of reactants is replaced by the sequential, alternating exposure of chemical precursors to form thin films. The surface reactions are self‐limiting in nature, thus allowing for precise control over film thickness at the atomic level and conformal deposition across complex surfaces.^[^ ^140^ ^]^ ALD was first reported by T. Suntola in Finland in 1974 through the development of ZnS films for electroluminescent displays, in a process referred to as atomic layer epitaxy (ALE).^[^ ^141^, ^142^ ^]^ It was also developed independently in Russia in the 1960s by S. Kol'tsov and V.B. Aleskovskii under the name molecular layering.^[^ ^143^ ^]^

**Figure 4:**
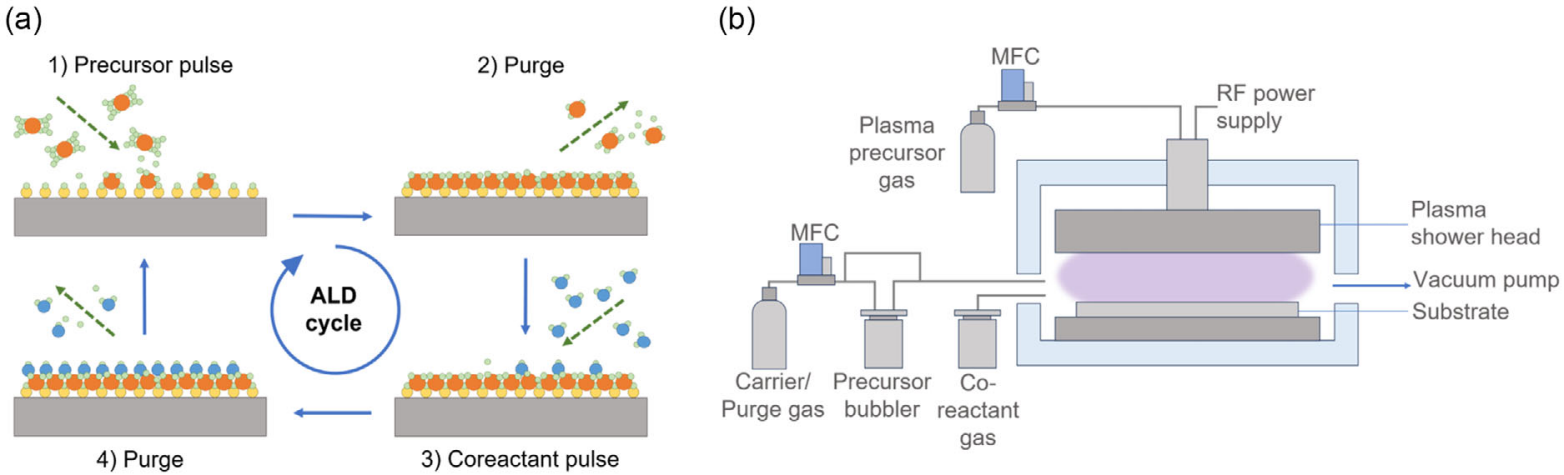
Schematic of a) typical ALD sequence composed of precursor and reactant pulse and purge cycle and b) PEALD reactor with RF power generator.

As illustrated in **Figure** **4**a, a typical ALD cycle consists of four steps: 1) pulse of the 1st precursor on the substrate to form a (sub)monolayer; 2) purge by an inert gas to remove the unreacted precursor and gaseous by‐products; 3) pulse of the 2nd precursor (reactant); and 4) purge of the excess precursor and by‐products to form the target film material.^[^ ^144^ ^]^ The growth rate of the ALD film, referred to as growth‐per‐cycle (GPC), is typically slower than conventional CVD processes.^[^ ^89^ ^]^ Each ALD process occurs at a specific temperature range, which is called the ALD window, where the GPC is nearly constant. Below this temperature window, the GPC may increase from reactant condensation on the surface or decrease due to insufficient thermal energy. Above this window, growth may increase from precursor decomposition, similar to CVD, or decrease due to desorption of surface species.^[^ ^145^, ^146^ ^]^ It should be noted that variations in deposition temperature can significantly affect film crystallinity, where higher temperatures often lead to improved crystallinity but may also induce undesirable roughness or grain growth, depending on the material system.^[^ ^147^, ^148^ ^]^

Although ALD is driven by sequential self‐limiting surface processes, many precursors used for the ALD of Cu films are based on precursors designed for MOCVD. Cu (II) precursors are often preferred for ALD as they are more stable than Cu (I) precursors, which tend to decompose easily upon contact with the substrate. In addition, the ALD process of Cu typically operates at a lower thermal limit than CVD, as higher temperatures can induce premature decomposition of the precursors. These lower deposition temperatures in ALD enable the formation of smoother, more uniform Cu films compared to CVD.^[^ ^149^ ^]^ Such limitations are apparent in CuCl, which requires high evaporation temperatures due to its low volatility. β‐diketonates and fluorinated precursors suffer from low reactivity and introduce carbon, oxygen, or fluorine impurities, which degrade film conductivity, smoothness, and adhesion. Amidinates offer improved hydrogen reactivity and film purity at lower temperatures, though their high melting points can cause inconsistent vapor delivery. Aminoalkoxides, in contrast, enable low‐temperature deposition of Cu with minimal nitrogen and oxygen contamination, offering a balance of volatility and reactivity for both CVD and ALD processes.^[^ ^116^, ^148^ ^]^

Like conventional CVD, the final composition of the ALD film depends on the nature of the reactant gases. For instance, using hydrogen gas as the reductant produces metallic Cu films. Diethyl zinc is also used as a reductant, although care is needed to avoid metallic zinc contamination^[^ ^150^, ^151^ ^]^ Alternatively, copper oxides form with O_2_, H_2_O, or O_3_ reactants, while copper nitrides and sulfides can be produced with NH_3_ and H_2_S, respectively.^[^ ^152^ ^]^ These Cu ALD processes, along with other metals, are summarized in review papers by Gordon et al.,^[^ ^116^ ^]^ George,^[^ ^146^ ^]^ Li et al.,^[^ ^152^ ^]^ and Miikkulainen et al.,^[^ ^153^ ^]^ as well as in the online ALD database atomiclimits.com.^[^ ^154^ ^]^ Several examples of different deposition conditions for ALD fabrication of Cu and Cu oxide films are shown in **Table** **4**.

**Table 4:** Examples of ALD of Cu‐based films.

Precursor	Reactant	Precursor temp [°C]	Deposition temp [°C]	Pressure [mbar]a)	Growth per cycle [Å]b)	Product	Substrate	Ref.
CuCl	H_2_/H_2_O	340	375–475	10.7	0.2–1.7	Cu	SiO_2_/Al_2_O_3_	[150]
Cu(OAc)_2_	H_2_O	175‐185	180–220	≈10	0.08 −0.14	Cu_2_O	Si	[186]
Cu(acac)_2_	H_2_ plasma	125	140	1	0.18	Cu	Si/SiO_2_	[187]
Cu(acac)_2_	H_2_O/O_2_	140	200	–	0.07	Cu_2_O	Si	[188]
Cu(dmap)_2_	H_2_O	100	110–175	–	0.12 ± 0.02	Cu_2_O	SiO_2_	[189]
Cu(dmap)_2_	DEZ	70	100–120	0.4	0.2	Cu	Si	[151]
[Cu(^s^Bu‐amd)]_2_	H_2_	–	150–190	–	0.15–0.2	Cu	SiO_2_/Si_3_N_4_	[148]
Cu(thd)_2_	H_2_	120	190–260	6.7–13.3	≈0.35	Cu	Glass/Pt/Pd	[190]

a) Deposition pressure values are converted to mbar unit;

b) Growth rate values are converted to Angstrom per cycle.

#### 3.3.1 Thermal and Plasma‐Enhanced ALD

The two most prevalent ALD process modes are thermal ALD and plasma‐enhanced ALD (PEALD). Thermal ALD, which is driven exclusively by surface reactions, ensures excellent thickness control and conformality regardless of substrate geometry and reactor design. However, its relatively high temperature requirements (150–350 °C) limit its applications.^[^ ^155^ ^]^ To decrease the deposition temperature, PEALD (Figure 4b) was developed with the use of plasma as a reactant. Plasma enables higher precursor reactivity, which shortens deposition time and forms denser films. However, this can result in reduced conformality and film damage.^[^ ^156^ ^]^

#### 3.3.2 Other ALD Processes

Specialized modes of ALD are also utilized for Cu film deposition. Spatial ALD uses fixed reaction zones with continuous precursor supply separated by inert gas barriers, where the substrate moves through each zone to mimic the temporal precursor delivery of conventional ALD. Atmospheric ALD supports deposition in open air, without requiring a sealed vacuum chamber, for low‐cost film fabrication.^[^ ^157^ ^]^ Area‐selective ALD allows film growth on specific substrate regions by using surface passivation (e.g., with self‐assembled monolayers or polymer films), functionalization to activate growth areas, or precursors that are inherently selective to certain substrates. Photo‐assisted ALD is a variant of area‐selective ALD, which uses targeted photon exposure to activate precursor reactions, without the need for surface treatments.^[^ ^158^ ^]^ Although not a vapor‐phase technique, electrochemical ALD (ECALD) achieves layer‐by‐layer growth using liquid‐phase precursors and controlled electrode potentials, offering a cost‐effective, scalable, and high‐throughput alternative to conventional ALD.^[^ ^159^ ^]^

### 3.4 Comparison of Vapor Deposition Techniques

#### 3.4.1 Deposition Mechanism

PVD deposits copper by physically ejecting atoms from a solid target under vacuum, resulting in line‐of‐sight growth that limits coverage on nonplanar or high aspect‐ratio substrates. In contrast, CVD uses volatile copper precursors that chemically react or decompose on the substrate surface, allowing better coverage of complex geometries. However, variations in film growth can still occur due to precursor depletion or uneven reaction kinetics. ALD offers the highest level of control by employing sequential, self‐limiting surface reactions, which enable atomic‐scale thickness control and uniform coatings even on substrates with intricate shapes or porosity.^[^ ^89^, ^116^, ^160^ ^]^

#### 3.4.2 Film Uniformity and Thickness Control

On flat surfaces, PVD can achieve relatively uniform films, but its inability to reach recessed areas limits its use for complex structures. CVD offers better uniformity than PVD due to gas‐phase transport, though it can still show variations in step coverage. ALD achieves highly uniform, conformal films regardless of substrate geometry, with each cycle depositing a controlled material amount, ensuring reproducibility and precise thickness control.^[^ ^89^ ^]^

#### 3.4.3 Process Temperature and Substrate Compatibility

The low operating temperature of PVD, often at RT for sputtering, makes it compatible with thermally sensitive substrates like polymers. However, because it relies on physical deposition without surface reactions, it offers limited chemical versatility. CVD typically requires higher process temperatures (up to 400 °C or more) to activate precursor chemistry, which can restrict its use with temperature‐sensitive materials and cause interdiffusion at interfaces. ALD typically operates in an intermediate range (100–200 °C), providing a favorable balance between precursor reactivity and substrate compatibility. For all processes, plasma can be integrated to decrease the deposition temperature. In CVD and ALD, the use of metal–organic precursors can introduce impurities such as carbon, oxygen, or nitrogen due to incomplete decomposition. PVD methods, while typically cleaner, can still suffer from contamination arising from target impurities, residual chamber gases, or unintended redeposition of sputtered species.^[^ ^106^, ^116^, ^161^ ^]^

#### 3.4.4 Scalability and Throughput

PVD is widely used due to its high deposition rates and straightforward implementation, making it useful for large‐area and high‐throughput applications. However, its effectiveness decreases with increasing structural complexity. CVD offers better conformality and scalability, especially when precursor flow and reaction conditions are carefully optimized. However, in the case of copper, CVD faces challenges such as precursor instability, carbon contamination, and process complexity, which limit its scalability and widespread industrial adoption. Nonetheless, it has been applied for producing nanostructured coatings or conformal copper films on complex surfaces. ALD, while traditionally limited by slower cycle‐based deposition, has seen significant advances in throughput through technologies such as spatial or batch‐type ALD. These developments have made it increasingly viable for applications where coating precision, conformality, and uniformity are more critical than speed.^[^ ^70^, ^116^, ^157^ ^]^

#### 3.4.5 Cost

The overall cost of deposition depends on factors like precursor pricing, equipment complexity, vacuum needs, and process efficiency. PVD benefits from relatively low precursor costs due to the use of solid copper targets, but its requirement for high‐vacuum systems and inefficiencies when coating complex surfaces can elevate operational expenses. CVD introduces higher costs associated with specialized precursors and the need for precise control over gas flow, temperature, and exhaust treatment, which also increases infrastructure and safety demands. ALD generally incurs the highest material and equipment costs because of its reliance on high‐purity precursors, multiple dosing cycles, and sophisticated process control. Despite these higher expenses, ALD's ability to deliver unmatched thickness precision and conformality often justifies the investment for applications demanding nanoscale accuracy.^[^ ^161^, ^162^ ^]^

**Table** **5** summarizes the differences between PVD, CVD, and ALD processes, detailing key characteristics such as film uniformity, conformity, growth rate, and film thickness.

**Table 5:** Comparison of PVD, CVD, and ALD.

Film deposition techniquea)	Deposition principle	Deposition pattern	Uniformity	Conformality	Deposition temperatureb)	Degree of vacuum	Growth rate	Film thickness
PVD	Physical vapor deposition	Nucleation growth	High number of pinholes and particles exist	Low and only for flat substrate	Low	High requirement for vacuum degree and sensitive to the change of vacuum degree	Fast	Nanometer level
CVD	Gas‐phase chemical reaction	Nucleation growth	Low number of pinholes and particles exist	Medium	High	Medium	Medium	Nanometer level
ALD	Surface saturated reaction	Layer‐by‐layer growth	Low number of pinholes and no particles	High	Medium	Low	Slow	Angstrom level

a) Modified from ref. [191]. Copyright Yu et al., 2024. This article is licensed under a Creative Commons Attribution 4.0 International License (https://creativecommons.org/licenses/by/4.0/);

b) Respective average temperature range for Cu film deposition—low (RT), medium (100–200 °C), and high (200–550 °C).

## 4 Vapor Deposition of Cu‐Based Electrocatalysts

### 4.1 Monometallic Copper Electrocatalysts

#### 4.1.1 Effect of Morphology

**Figure 5:**
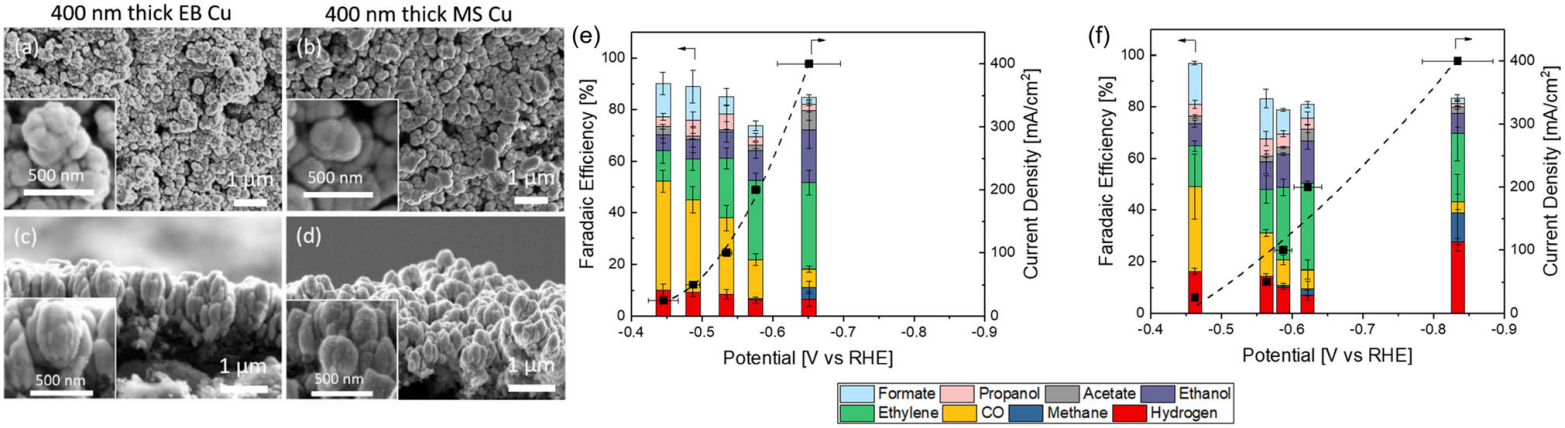
Scanning electron microscopy (SEM) images of a) 400 nm thick EB and b) MS‐Cu coatings on GDLs, and c,d) respective cross‐sectional SEM images. FEs and current densities versus potential for CO2RR for 400 nm e) EB‐Cu and f) MS‐Cu catalysts using three‐compartment flow cell with 15 sccm CO_2_ and 1 m KOH electrolyte. Adapted with permission.^[^ ^163^ ^]^ Copyright 2022, American Chemical Society.

The morphological characteristics of Cu electrocatalysts, including surface roughness, particle distribution, film uniformity, and crystallographic facet exposure, can affect local reaction environments and CO2RR product selectivity. Jeng et al.^[^ ^163^ ^]^ conducted direct current MS and EB deposition to coat Cu catalysts onto GDLs. While both coatings contain similarly sized Cu particles, the EB‐Cu displays more well‐defined facets, in contrast to MS‐Cu, which exhibits a rough and serrated appearance (**Figure** **5**a–d). Microfluidic flow cell measurements with 15 sccm (standard cubic centimeters per minute) CO_2_ and 1 M KOH revealed a similar CO2RR product spectrum for both catalysts (Figure 5e,f), but notable differences in selectivity at high current density (− 400 mA cm^−^^2^): EB‐Cu achieved 70% FE for C_2+_ products, while MS‐Cu showed enhanced FE for CH_4_% and 28% FE for H_2_. The MS‐Cu sample also required higher overpotentials to achieve the same current density values as the EB‐Cu sample. This poor suppression of H_2_ at high overpotential for MS‐Cu was attributed to its more hydrophilic nature, which is related to its rougher nanoscale surface morphology.

**Figure 6:**
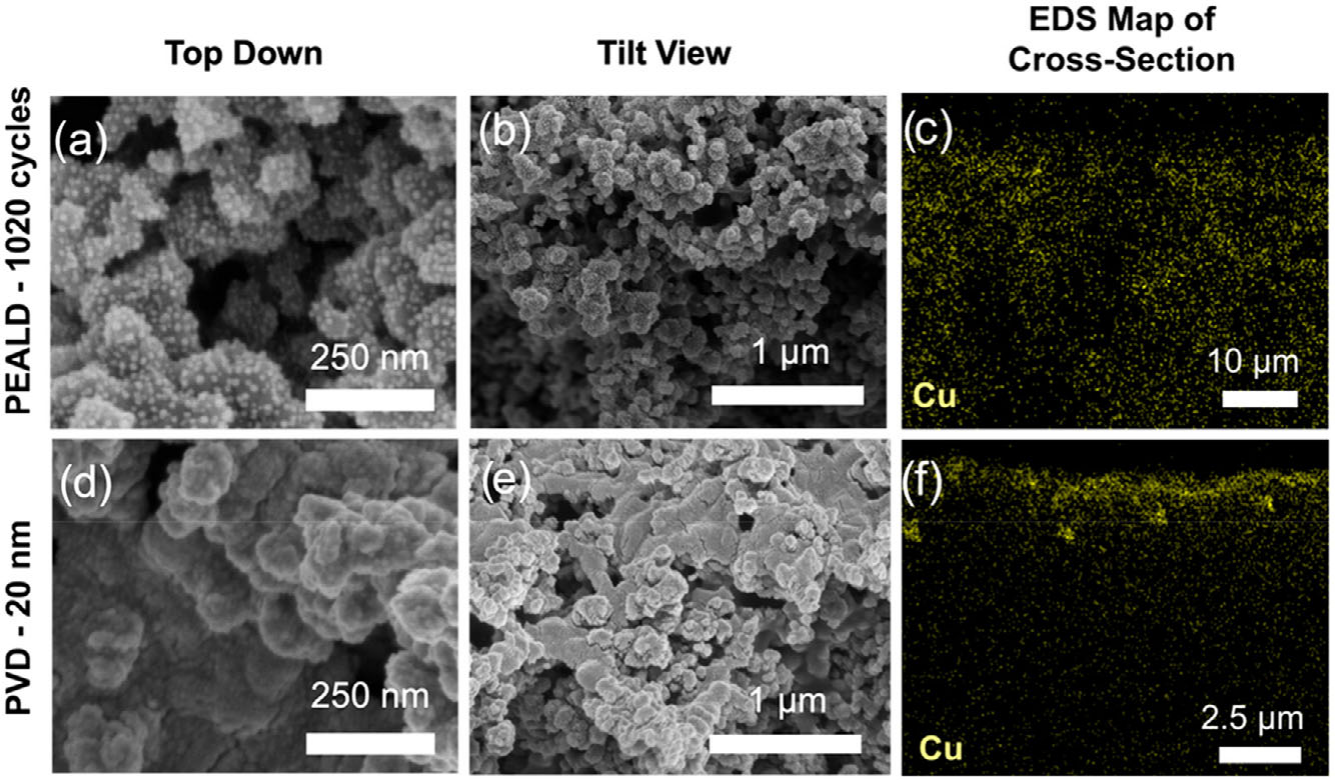
SEM of a–c) PEALD Cu with 1020 cycles and d–f) 20 nm PVD Cu: (a,d) top down, (b,e) tilt view, and (c,f) EDS Cu cross‐section map of the microporous layer. Adapted with permission.^[^ ^160^ ^]^ Copyright 2023 American Chemical Society.

Lenef et al.^[^ ^160^ ^]^ compared PEALD and PVD‐EB evaporation methods in the fabrication of Cu GDEs. The PEALD samples were made using Copper(I) N,N′‐disec‐butylacetamidinate ([Cu(^s^Bu‐amd)]_2_) precursor and H_2_ plasma reductant at 185 °C, achieving a growth rate of ~0.2 Å per cycle, while PVD samples were deposited at 5 Å per second. The PEALD‐Cu sample (1020 cycles) exhibited a discrete nanoparticle morphology with deep and uniform conformance to the microporous layer of GDLs, preserving the porosity for effective mass transport and higher catalyst/electrolyte contact area (**Figure** **6**a–c). On the other hand, the PVD sample (20 nm) formed dense and continuous particles that clog the GDL surface, reducing the depth of infiltration and decreasing the porosity (d–f). At −0.97 V versus RHE, the PEALD‐Cu has three times the current density and mass activity of PVD‐Cu and an improved FE for C_2+_ products of 28.4%. These results highlight the advantage of PEALD in particle uniformity and conformance, which is highly suitable for CO2RR applications.

**Figure 7:**
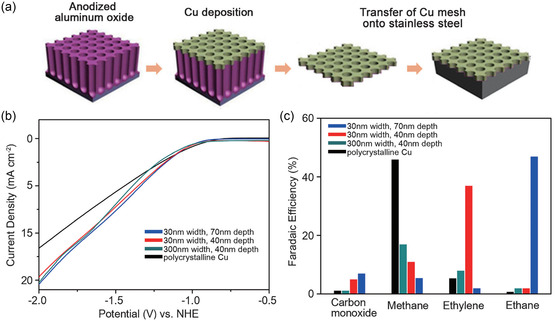
a) Scheme for preparing Cu mesopore electrodes. b) Linear sweep voltammetry curves and c) CO2RR product selectivity of the Cu mesopore electrodes at −1.7 V versus NHE using H‐cell with 0.1 m KHCO_3_ (CO_2_ saturated). Adapted with permission.^[^ ^164^ ^]^ Copyright 2017, Wiley‐VCH Verlag GmbH & Co. KGaA, Weinheim.

Mesoporous Cu electrodes with tunable selectivity can be fabricated by controlling pore diameter and depth through sputtering. Yang et al.^[^ ^164^ ^]^ employed direct current sputtering to deposit Cu onto anodized aluminum oxide templates, creating three Cu meshes with varying mesopore dimensions (width/depth). These meshes were attached to stainless steel to form electrodes (**Figure** **7**a) for CO_2_ electroreduction in an H‐cell. All electrodes displayed similar current densities of ≈− 14.3 mA cm^−^^2^ at − 1.7 V versus the normal hydrogen electrode (NHE), yet their product selectivity of each varied significantly under the same overpotential (Figure 7b,c). The 300 nm/40 nm electrode favored C_1_ production with 18% FE_CH4_% and 32% FE_HCOOH_. In contrast, the narrower 30 nm/40 nm and 30 nm/70 nm electrodes favored C_2_ production, with 38% FE_C2H4_% and 46% FE_C2H6_, respectively. Supported by electrohydrodynamic simulations, the study proposed that the mesopore dimensions affect the local pH and retention time of reaction intermediates inside the pores, leading to variations in product selectivity.

Reactive magnetron sputtering can be utilized for facet‐engineering of Cu electrodes that are highly scalable. Zhang et al.^[^ ^70^ ^]^ varied RF power to tune the kinetic energy of the bombarding Cu atoms, yielding high power sputtered Cu (200 W RF power) which favors Cu(100) facets and low power sputtered Cu (40 W RF power) which favors Cu(111) facets. To increase the electrochemically active surface area (ECSA) of these catalysts, an oxidation‐reduction step was performed through the introduction of molecular O_2_ during sputtering followed by an electroreduction step, while retaining the Cu(100) facets exposure. The high‐power reactively sputtered Cu films (HRS‐Cu) achieved a maximum FE for C_2_H_4_ at 58.6% and a stability of 4.5 h at − 0.75 V versus RHE, using a flow cell with 2 m KOH. The HRS‐Cu was scaled‐up using a 25 cm^2^ MEA which maintained an FE for C_2_H_4_ above 45% after 3.5 h operation at 12 A total current. In situ attenuated total reflectance–surface‐enhanced infrared absorption spectroscopy (ATR‐SEIRAS) reveals that higher Cu(100) facet exposure increases *CO coverage, promoting C—C coupling and formation of C_2+_ products, while lower Cu(100) leads to lower *CO coverage, higher *H coverage, and a shift in selectivity toward C_1_ products like methane.

#### 4.1.2 Effect of Thickness

**Figure 8:**
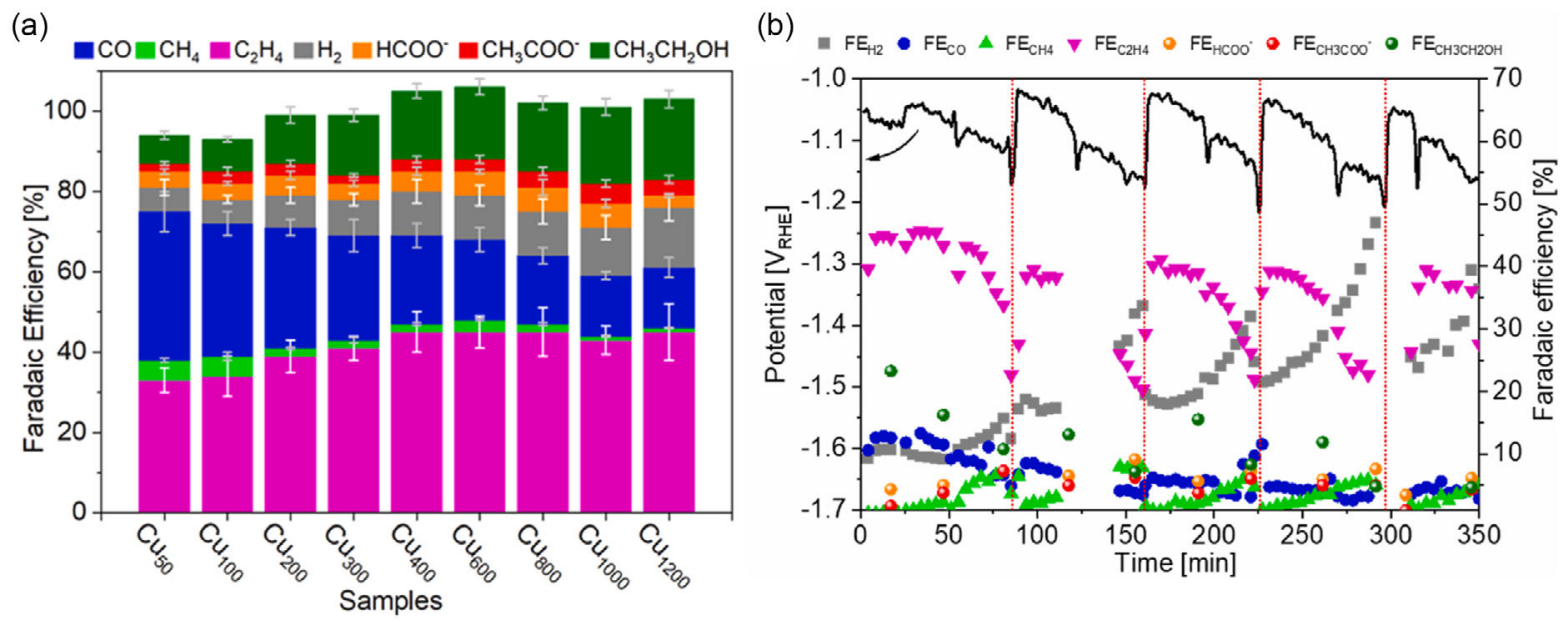
a) CO_2_RR tests on sputtered Cu GDEs of varying thickness at −1.2 V versus RHE. b) Long‐term test of Cu800 at 200 mA cm^−2^ with pulsed electrolysis. Figure adapted from ref. [165] licensed under Creative Commons Attribution 4.0 International (CC BY 4.0) (https://creativecommons.org/licenses/by/4.0/).

The effect of catalyst layer thickness on the stability of magnetron sputtered Cu electrodes was examined by Monti et al.^[^ ^165^ ^]^ Various Cu GDEs were fabricated by increasing the deposition time from 50 to 1200 s, at a fixed 50 mA current, with an observed growth rate of 0.59 nm s^−^^1^. Using a flow cell with 1.0 m KOH and a fixed potential of − 1.2 V versus RHE, it was observed that the C_2_H_4_ selectivity increases with Cu layer thickness, reaching a plateau from 400 s sputtering time, while CO selectivity decreases with increasing thickness (**Figure** **8**a). The trend is attributed to longer retention time of CO, a key intermediate for C_2_H_4_ formation, in thicker catalyst layers. Increasing the catalyst thickness also improves electrode stability, with the Cu600 and Cu800 electrodes maintaining above 40% C_2_H_4_ selectivity for over an hour, and even longer durations on Cu1000 and Cu1200 electrodes. The enhanced stability was associated with higher catalyst loading and surface area, which slows down catalyst restructuring and delays flooding or salt‐induced choking of the electrodes. It was also emphasized that high electrolyte pH compromises electrode stability, as Cu electrodes tend to become unstable under such conditions.

A more detailed analysis of Cu800 at 200 mA cm^−^^2^ revealed that C_2_H_4_ and ethanol selectivity remained stable near 40% and 20%, respectively, for nearly an hour before declining due to electrode failure likely caused by flooding and salt deposition (Figure 8b). The time before this selectivity switch was correlated with catalyst thickness or loading, possibly reflecting differences in oxide content and salt accumulation. In addition, periodic interruptions of electrolysis by switching to open‐circuit conditions were shown to partially restore electrode performance up to 6 h, suggesting that managing the catalyst's local environment can improve stability during long‐term operation.

### 4.2 Bimetallic Copper Electrocatalysts

Bimetallic Cu‐based electrocatalysts have been synthesized using diverse vapor deposition approaches, such as codeposition, layering, coating, and composite target techniques. Each method offers distinct advantages in controlling catalyst composition, morphology, and surface properties, ultimately influencing catalytic performance.

#### 4.2.1 Codeposition Approach

A magnetron cosputtering approach was performed by Liu et al.^[^ ^166^ ^]^ to fabricate copper‐gold bimetallic electrodes with improved selectivity to CO. Using RF power for the Au target and DC power for the Cu target, AuCu films of different atomic ratios were deposited onto Ti foil by varying the sputtering powers of both targets. The films possessed a smooth and uniform morphology with clear evidence of Au—Cu alloying across all compositions. Electrochemical H‐cell measurements showed a maximum current density of −4.0 mA cm^−^^2^ for the AuCu catalysts. Increasing the Au content enhanced CO selectivity while reducing HCOO^−^ selectivity, with Au_75_Cu_25_ achieving over 60% FE_CO_ and 2% FE_HCOO‐_ at −0.7 V versus RHE. The authors proposed that Au can weaken the binding strength of the *CO intermediate, as evidenced by the d‐band center shifting away from the Fermi level and lower CO coverage from CO stripping experiments. Moreover, the d‐band center shifts also correspond to weaker oxygen binding strengths, limiting the formation of *OCHO which strongly influences HCOO^−^ production. Li et al.^[^ ^167^ ^]^ and Van der Veer et al.^[^ ^168^ ^]^ investigated Cu—Ag co‐sputtered electrodes, reporting enhanced ethanol FE compared to pure Cu which is more selective to C_2_H_4_. This was associated with the homogeneous distribution of Ag and Cu for cosputtered electrodes, as Ag introduces diverse binding sites with lower carbon affinity to partially shift the selectivity from ethylene to ethanol.

**Figure 9:**
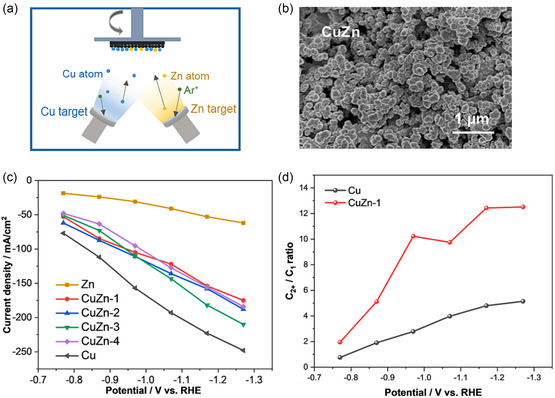
a) Schematic of the magnetron cosputtering deposition of the CuZn electrodes. b) SEM image of the CuZn sample. c) Total current density of Cu, Zn, and various CuZn electrodes and d) FE ratio of C_*2+*_/C_*1*_ products for Cu and CuZn‐1 electrodes using GDE‐based electrolytic cell with 20 sccm CO_2_ and 1M KOH. Adapted with permission.^[^ ^110^ ^]^ Copyright 2023, Elsevier B.V.

Yang et al.^[^ ^110^ ^]^ prepared various Cu—Zn GDEs by cosputtering pure Cu at constant power and Zn at varying power yielding CuZn‐1, CuZn‐2, CuZn‐3, and CuZn‐4 samples with Zn atomic percentage values of 10%, 46%, 57%, and 63%, respectively, as determined by EDS measurements. From the SEM image (**Figure** **9**b), the cosputtered catalysts exhibit uniform and evenly dispersed clusters of CuZn film. In terms of electrochemical performance, the CuZn samples demonstrated lower current densities than the pure Cu sample (Figure 9c). However, the CuZn‐1 sample has higher selectivity to C_2+_ products over a wide range of potentials than the pure Cu sample (Figure 9d). From in situ‐Fourier transform infrared spectroscopy (FTIR) and density functional theory (DFT) results, the minute addition of Zn to Cu has been found to optimize the adsorption energy of *CO intermediates, thereby promoting the formation of C_2+_ products through C—C dimerization.

Lee et al.^[^ ^169^ ^]^ demonstrated a complementary approach to synthesize Cu—Zn catalysts by combining thermal ALD of ZnO and PEALD of Cu, the latter as described by Lenef et al.^[^ ^160^ ^]^ Alternating supercycles of diethyl zinc (DEZ) and water for ZnO and Cu(^s^Bu‐amd)]_2_) and H_2_ plasma for Cu, allowed precise control of the Cu:Zn ratio. While the PEALD Cu GDE is selective to C_2_H_4_, with an FE of ≈38% at − 0.93 V versus RHE, the 9:1 Cu—Zn catalyst has reduced C_2_H_4_ selectivity to below 3%, and increased FE to CO by ≈31% at −0.90 V versus RHE. By increasing the Cu:Zn cycle ratio to 81:1, the Zn content was lowered, resulting in suppressed CO and methane production and enhanced selectivity toward C_2_H_4_, demonstrating that lower Zn incorporation can improve overall catalyst activity and multi‐carbon product selectivity.

#### 4.2.2 Overcoating Approach

Lee et al.^[^ ^169^ ^]^ additionally applied ALD to deposit thin ZnO overcoating layers on the PEALD Cu electrodes. Remarkably, even a single cycle of ZnO (≈2 Å thick) overcoating significantly suppressed C_2_H_4_ formation to 9.8% and enhanced CO production o 21% at −0.93 V versus RHE. With further ZnO deposition (3 cycles), C_2_H_4_ selectivity decreased to 4.2%, with a slight increase in CO FE to 22.5%. These selectivity trends were consistent across various applied potentials, while the current density remained largely unchanged. This suggests that the initial ZnO deposition induces the most substantial modification of the catalyst surface, while additional ZnO layers yield progressively smaller effects.

**Figure 10:**
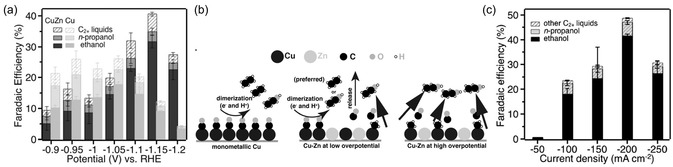
a) FE of C_2+_ liquid products of Cu and CuZn catalysts versus applied potential in an H‐cell using 0.1 m KHCO_3_. b) Proposed mechanism of ethanol formation of Cu and CuZn catalysts. c) FE of liquid products at selected current densities of CuZn in an electrochemical flow cell using 1M KOH. Adapted with permission.^[^ ^170^ ^]^ Copyright 2019, Wiley‐VCH Verlag GmbH & Co. KGaA, Weinheim.

A similar method was developed by Ren et al.^[^ ^170^ ^]^ by the ALD of ZnO on CuO nanowires. The CuO electrodes were fabricated by Cu magnetron sputtering, anodization, and annealing to form CuO nanowires (100 nm) on GDL substrates. Then, ZnO was deposited through 100 ALD cycles (30 nm) using DEZ and water at 120 °C. Prior to CO_2_ reduction, the CuO and CuO/ZnO electrodes were electrochemically reduced to Cu and CuZn, respectively. The H‐cell tests in 0.1 m KHCO_3_ revealed that CuZn has a higher selectivity and activity for ethanol compared to Cu at potentials below − 1.10 V versus RHE (**Figure** **10**a). Operando Raman measurements and partial current density analysis revealed that Zn modifies the binding sites of Cu to enhance CO formation. As the *CH_3_ intermediate concentration increases at more negative potentials, CO is more likely to couple with *CH_3_ to form the *COCH_3_ intermediate for ethanol production (Figure 10b), whereas CO dimerization is proposed as a pathway for ethanol formation on monometallic Cu. In flow‐cell tests with 1 M KOH, CuZn achieved good selectivity to C_2+_ liquids, with an FE of 48.6% at a current density of − 200 mA cm^−^^2^ at − 0.68 V versus RHE (Figure 10c).

#### 4.2.3 Layering Approach

Li et al.^[^ ^167^ ^]^ also compared layered Cu‐Ag electrodes made by sequential magnetron sputtering to cosputtered ones. The layered electrodes had a lower FE for ethanol (max 26%) than the cosputtered ones (max 41%), which was attributed to their less homogeneous metal distribution and fewer diverse binding sites. Due to the lack of uniform Ag and Cu mixing, the layered structures formed clusters of Ag and Cu which behave similarly to their bulk counterparts.

Van der Veer et al.^[^ ^168^ ^]^ further investigated the effect of layering order of the Cu—Ag samples. When Cu was sputtered first (Cu_80_Ag_20_), CO_2_ initially contacted the Cu surface, favoring C_2+_ product formation similarly to pure Cu, while unreacted CO_2_ subsequently reached the Ag layer, leading to an increase in CO generation (from 7.3 to 18.1%) and a slight reduction in C_2+_ FE (from 73.5% to ≈66%) compared to pure Cu. In contrast, sputtering Ag first resulted in substantially higher CO formation (up to 28.3% at Ag_20_Cu_80_) and a pronounced decline in C_2+_ selectivity (<50%). This behavior was attributed to limited CO spillover from the Ag layer to the overlying Cu, causing a significant portion of CO to diffuse out of the system rather than undergoing further reduction to C_2+_ products.

#### 4.2.4 Composite Target Approach

**Figure 11:**
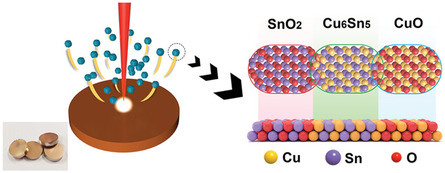
Schematic of pulse laser sputtering of CuSn sheet to form SnO_2_/Cu_6_Sn_5_/CuO tandem electrocatalyst. Adapted with permission.^[^ ^171^ ^]^ Copyright 2023, Wiley‐VCH Verlag GmbH & Co. KGaA, Weinheim.

Shi et al.^[^ ^171^ ^]^ developed a composite target strategy using pulsed laser sputtering for fabricating Cu‐ and Sn‐based tandem electrocatalysts with multicomponent CO2RR active sites. Unlike conventional cosputtering that requires separate targets for each metal, this approach used custom‐made targets prepared by mixing ground Cu and Sn powders in different molar ratios and pressing them into metal sheets with a metallic luster. Using a combination of oxidizing atmosphere (20% O_2_% and 80% Ar) and high temperature (700 °C) during laser sputtering, SnO_2_/Cu_6_Sn_5_/CuO nanocatalysts with superscalar phase boundaries were successfully fabricated. Flow cell tests demonstrated a high FE_HCOOH_ of 95.64% at −0.95 V versus RHE and current density of −70 mA cm^−^^2^ using 25 sccm CO_2_ and 1 m KOH. The predominant formic acid formation was attributed to the tandem effect of the catalysts, where SnO_2_ improved CO_2_ adsorption and activation, while CuO promoted H_2_O decomposition and increased proton supply, allowing *H to react with *CO_2_^−^ on Cu_6_Sn_5_ to form formic acid. **Figure** **11** illustrates the step‐by‐step diagram of CO2RR catalytic mechanism on the electrode.

### 4.3 Single‐Atom Catalysts

**Figure 12:**
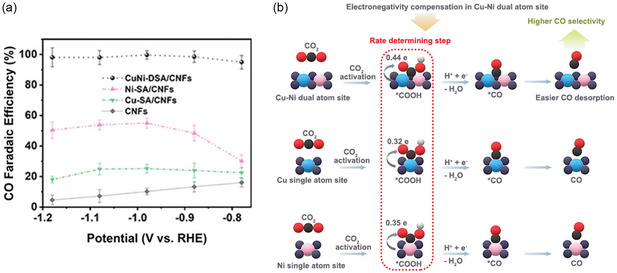
a) FE for CO and b) comparison of CO_2_RR pathways for Cu—Ni DSA, Cu‐SA, and Ni‐SA catalysts. Adapted with permission.^[^ ^172^ ^]^ Copyright 2019, Wiley‐VCH Verlag GmbH & Co. KGaA, Weinheim.

Through CVD, Hao et al.^[^ ^172^ ^]^ synthesized Cu single atom (Cu‐SA), Ni single atom (Ni‐SA), and Cu‐Ni dual‐single atom (CuNi‐DSA) catalysts onto carbon nanofibers (CNFs). In a CVD furnace, CuCl_**2**_ and NiCl_**2**_ salts and polyvinyl pyrrolidone (PVP) electrospun NF membranes underwent graphitization at 1000 °C for 3 h, during which Cu and Ni vapors diffused through the PVP‐derived CNFs and were trapped to form CuNi‐DSA/CNFs, where Cu and adjacent Ni atoms are each coordinated with four nitrogen atoms (CuN_**4**_–NiN_**4**_). The Cu‐SA/CNFs and Ni‐SA/CNFs were prepared similarly using only CuCl_**2**_ and NiCl_**2,**_ respectively. In a 0.1 M KHCO_3_ H‐cell, CuNi‐DSA/CNFs achieved a maximum FE_CO_ of 99.6% at −0.98 V versus RHE (**Figure** **12**a), significantly outperforming Cu‐SA/CNFs (20.6% at −1.08 V), and Ni‐SA/CNFs (42.8% at −0.98 V). CuNi‐DSA/CNFs also show excellent stability, maintaining FE_CO_ above 98% for 25 h at −20 mA cm^−^^2^, and high performance in a 1 M KOH flow‐cell with FE_CO_ of 98.2% at −0.70 V and −80 mA cm^−^^2^. The superior CO selectivity of CuNi‐DSA/CNFs arises from the charge offset between Cu and Ni sites that promotes the formation of the *COOH intermediate, a key step in CO production, as supported by in situ Raman and DFT, while Cu‐SA and Ni‐SA show minimal *COOH coverage and lower activity (Figure 12b).

Cu single‐atom catalysts were also synthesized via ALD. Zhang et al.^[^ ^76^ ^]^ deposited Cu SACs onto Al_2_O_3_, CeO_2_, and TiO_2_ supports using Cu(hfac)_2_ and H_2_O at 280 °C, followed by heating at 480 °C to anchor the Cu atoms, after which the catalysts were drop cast onto GDEs. At − 400 mA cm^−^^2^, the CeO_2_‐Cu_SAC_ exhibited the highest CH_4_ selectivity of 70.3%, owing to the optimized electronic metal–support interaction (EMSI) between the Cu and CeO_2_ that balances CO_2_ activation and H_2_O protonation, promoting efficient methane formation while suppressing competing reactions. In contrast, the TiO_2_ support enhances H_2_O activation, intensifying the competing HER which limits CH_4_ selectivity. Meanwhile, the Al_2_O_3_‐Cu_SAC_ EMSI favors CO adsorption and weakens C—O bonds, which facilitates C—C coupling toward the formation of multicarbon species. By increasing the Cu precursor pulse from 50 ms to 3 s, Cu NPs were deposited onto Al_2_O_3_ support. The Al_2_O_3_‐CuNPs exhibited a stability of 70 h with FE_C2_ above 62%, while the CeO_2_‐Cu_SAC_ also showed 70 h stability with FE_CH4_ above 52%. This demonstrates the precise control of ALD over Cu dispersion to tailor catalyst design and optimize selectivity.

### 4.4 Modified Cu Catalysts

#### 4.4.1 Chemical Modification

**Figure 13:**
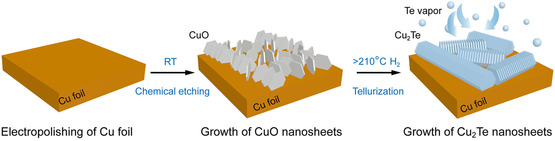
Schematic of the CVD of Cu_2_Te nanosheets. Adapted with permission.^[^ ^174^ ^]^ Copyright 2023, American Chemical Society.

The CVD technique has been used to incorporate secondary elements into Cu substrates. For example, Dřínek et al.^[^ ^173^ ^]^ fabricated Cu_*x*_Si catalysts through the CVD of butylsilane (BuSiH_3_) onto a commercial Cu sheet using a quartz tube oven at 500 °C. This process resulted in unique structures with large surface areas, such as microwires, nanowires, nanoribbons, and nanoplatelets. A particular sample with a [Cu]:[Si] ratio of 1.8 consisted of microwires with flat interwoven nanoribbons. Electrochemical testing in an H‐cell showed that this catalyst reached a maximum FE of 79% for ethanol at −0.50 V versus RHE using CO_2_‐saturated 0.1 m K_2_CO_3_, and a maximum of 72% FE for acetate at −0.65 V versus RHE using Ar‐saturated 0.1 m K_2_CO_3_. The authors attributed this shift in selectivity from ethanol to acetate due to the effect of electrolyte pH from neutral to basic conditions. A similar approach was reported by Wang et al.^[^ ^174^ ^]^ with the CVD of Cu_2_Te electrocatalysts for CO_2_ methanation. In a CVD furnace, Te powder was heated to ≈450 °C while etched Cu foil substrates were heated to 220–280 °C under a flow of H_2_ gas to form edge‐oriented 2D single‐crystal Cu_2_Te nanosheet arrays (**Figure** **13**). The formed Cu_2_Te powders were spray coated onto GDL for flow‐cell CO2RR tests, which demonstrated a ≈63% FE for CH_4_ at 300 mA cm^−^^2^. ECSA measurements, DFT calculations, and in situ ATR‐FTIR spectroscopy reveal that edge‐oriented Cu_2_Te nanosheets enhance CH_4_ selectivity by lowering the energy barrier for the rate‐limiting *CO to *CHO step.

Suominen et al.^[^ ^175^ ^]^ used ALD to form transition metal chalcogenide catalysts such as CuS_*x*_. Copper acetylacetonate (Cu(acac)_2_) and copper bis(2,2,6,6‐tetramethyl‐3,5‐heptanedionate) (Cu(thd)_2_) were used as precursors, with H_2_S as the reactant to produce Cu_2_S and CuS films, respectively. These films were deposited on ozone‐modified single‐walled carbon nanotubes (SWCNT‐O_3_) on GDL, with a ≈1 nm Al_2_O_3_ adhesion layer via 10 cycles of ALD using tetramethyl aluminum (TMA) and H_2_O. Flow cell tests revealed that the optimum electrode configuration consisted of 0.03 mg cm^−^^2^ of SWCNT‐O_3_, ≈1 nm Al_2_O_3_ adhesion layer, and ≈10 nm CuS_*x*_ layer, which achieved a FE_HCOO−_ of 60% at −0.76 V versus RHE using 0.1 M KHCO_3_. To elucidate the role of S in enhancing formate selectivity, a reference metallic Cu film was deposited on GDL through 500 cycles of ALD of copper bis(dimethylamino‐2‐propoxide) (Cu(dmap)_2_) with tert‐butyl hydrazine.^[^ ^147^ ^]^ In 0.1 m KHCO_3_ at −0.85 V versus RHE, these metallic Cu GDEs generated a high FE for H_2_ of almost 40%. Literature suggests that sulfur in Cu promotes CO* binding while leaving the H* sites unblocked, preventing the formation of CO and allowing the reaction of H* with CO_2_ to form formate.^[^ ^176^ ^]^

#### 4.4.2 Surface Modification

**Figure 14:**
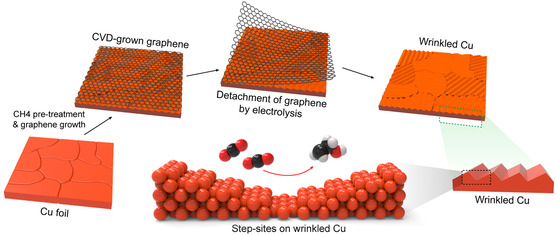
Schematic of the synthesis method for wrinkled Cu catalysts through CVD of graphene onto Cu foil, and graphene detachment via electrolysis. Adapted with permission.^[^ ^177^ ^]^ Copyright 2021, American Chemical Society.

Another innovative application of CVD lies in the surface engineering of Cu catalysts. Kim et al.^[^ ^177^ ^]^ applied CVD to grow a graphene monolayer onto Cu foil using CH_4_ gas in a quartz tube furnace at 1000 °C. Following graphene detachment by electrolysis, the Cu surface transformed into a wrinkled morphology with a high density of step‐sites and high facet atomic arrangement (**Figure** **14**). High‐resolution transmission electron microscopy (HR‐TEM) revealed that the initially flat Cu foil with (111) atomic arrangement likely underwent surface reconstruction into (200), (210), and (310) facets during the formation of the wrinkled Cu catalyst. These facets are typically challenging to obtain using conventional methods. Through CO2RR tests in an H‐cell, the wrinkled Cu exhibited a significantly high selectivity to ethanol of 40% at −0.9 V versus RHE with 0.1 m KCl, compared to only ≈5% for the flat Cu film under identical conditions. The supporting DFT calculations attributed the increased ethanol selectivity to the (310) facet, which has a low C—C coupling barrier and a favorable reaction pathway toward ethanol than other products.

**Figure 15:**
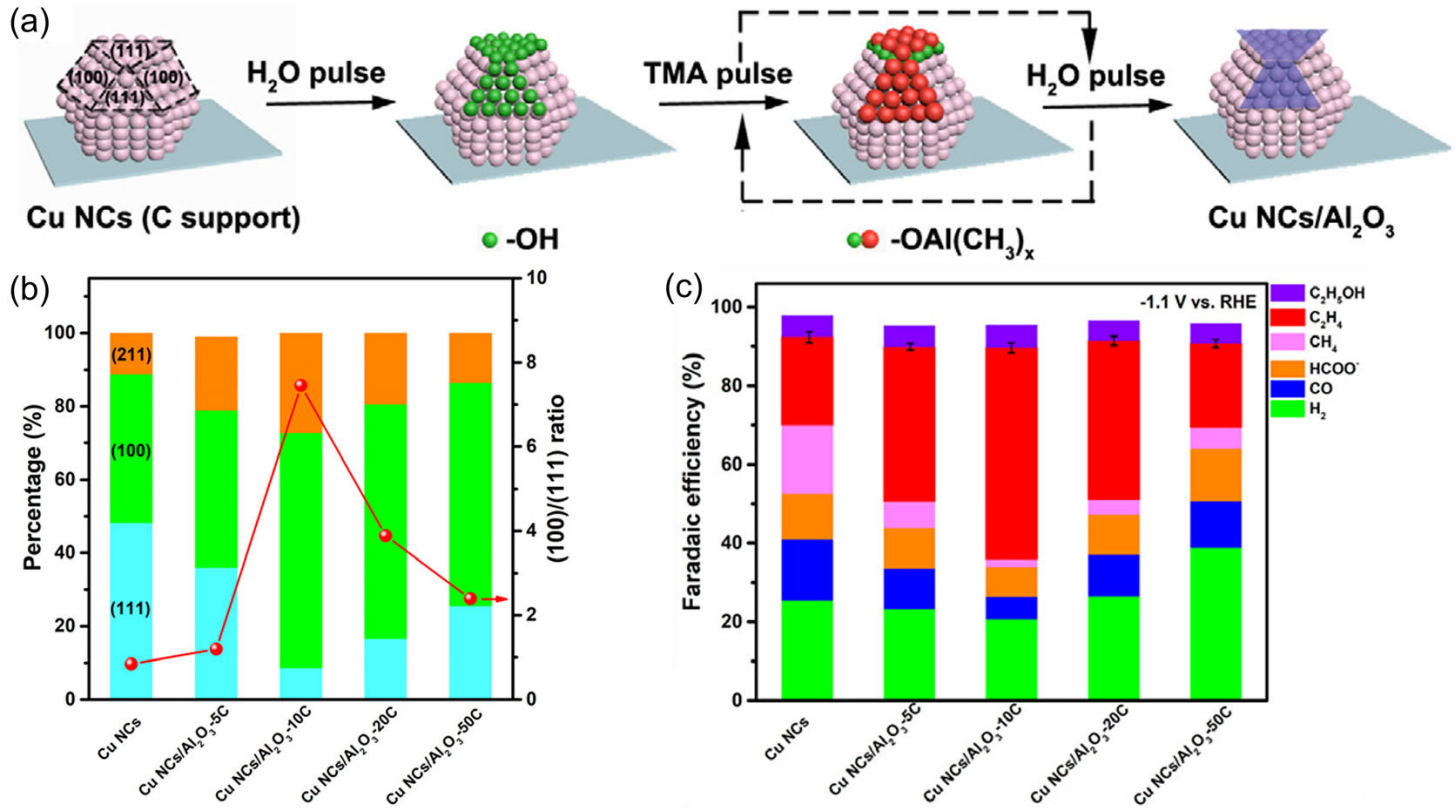
a) Schematic diagram of FSALD of Al_2_O_3_ on Cu nanocrystals. b) Facet percentage and Cu(100)/(111) facet ratio from diffuse reflectance infrared Fourier transform spectroscopy (DRIFTS) of CO chemisorption. c) FE at −1.1 V versus RHE of Cu NCs at varied ALD cycles of Al_2_O_3_ overcoating with an H‐cell in 0.5 m KHCO_3_. Adapted with permission.^[^ ^179^ ^]^ Copyright 2021, Wiley‐VCH Verlag GmbH & Co. KGaA, Weinheim.

ALD has been used for facet‐selective coatings of Cu electrodes, enabling the passivation or activation of specific sites through the varying binding energies of precursors on different facets.^[^ ^178^ ^]^ Li et al.^[^ ^179^ ^]^ performed ALD of Al_2_O_3_ to coat the Cu(111) facets of Cu nanocrystals (NCs), thereby enhancing Cu(100) facet exposure and improving C_2+_ product selectivity. Ultrathin Al_2_O_3_ coatings were deposited on carbon‐supported Cu NCs through alternating H_2_O‐TMA pulses, with the cycle number varied (**Figure** **15**a). The initial H_2_O pulse was essential to form hydroxide groups on the Cu(111) facets, facilitating preferential nucleation of TMA. As ALD cycles increased from 5 to 10, preferential blocking of the Cu(111) facets was observed, and further cycles led to blocking of the Cu(100) facets varied (Figure 15b). Thus, 10 ALD cycles (10C) were optimal for maximizing the Cu(100)/(111) facet ratio. The CO2RR tests in a H‐cell with 0.5 M KHCO_3_ indicate that the 10C sample exhibits the highest selectivity for C_2_H_4_ with an FE of 53.8%, 2.4 times higher than the bare Cu NCs (Figure 15c), while flow cell tests in 5 m KOH resulted in a higher FE_C2H4_ of 60.4% at −300 mA cm^−^^2^ with minimal selectivity decrease over 24 h. These results imply that increasing the (100)/(111) Cu facet ratio significantly promotes the formation of C_2_H_4_, consistent with various literature.^[^ ^70^, ^180^ ^]^ Furthermore, in situ small‐angle X‐ray scattering measurements show that the Al_2_O_3_ coating enhances the stability of the Cu NCs, as evidenced by the minimal changes in the mean size diameter and relative number density of the NCs after electrolysis.

## 5 Summary and Outlook

The use of vapor deposition techniques is an emerging strategy for the fabrication of Cu‐based electrocatalysts for electrochemical CO_2_ reduction. These methods, already well‐established for the commercial production of Cu thin films in the electronics industry, offer valuable expertise that can accelerate the development of highly pure and well‐controlled Cu‐based electrocatalysts, particularly for the preparation of gas diffusion electrodes. This work provides a comprehensive discussion of the primary vapor deposition techniques such as PVD, CVD, and ALD, focusing on their unique advantages, limitations, and potential toward the design and fabrication of high‐performance Cu‐based electrocatalysts and electrodes. A comparative summary of key results is provided in **Table** **6**.

**Table 6:** Summary of Cu‐based electrocatalysts developed by PVD, CVD, and ALD.

Catalyst	Method	Electrochemical reactor type	Current density [mA cm^−2^]	Potential (V versus RHE)	Product (FE)	Stability [h]	Electrolyte	Ref.
Cu 400 nma)	Electron beam evaporation of Cu on GDL	Flow cell	−400	−0.65 V	C_2+_ (70%), C_2_H_5_OH (≈20%), H_2_ (7%)	–	1 m KOH	[163]
Cu 400 nma)	Magnetron sputtering of Cu on GDL	Flow cell	−400	−0.83 V	C_2+_ (≈40%), C_2_H_5_OH (≈10%), H_2_ (28%)	–	1 m KOH	[163]
Cu 400 nma)	Sputtering of Cu on GDL	Flow cell	−150	−2.52 V (versus Ag/AgCl)	C_2_H_5_OH (22.8%), C_2_H_4_ (44.0%)	1	0.5 m KHCO_3_	[168]
Cu 800sb)	Magnetron Sputtering of Cu on GDL	Flow cell	−200	−1.1 V	C_2_H_4_ (41%)	6	1 m KOH	[165]
Cu(100)‐rich	High‐power reactive sputtering of Cu on GDL	Flow cell	≈ −120	−0.75 V	C_2_H_5_OH (58.6%)	4.5	2 m KOH	[70]
Cu mesopores	Sputtering of Cu (30 nm width/70 nm depth) on Al_2_O_3_	H‐cell	−14.3	−1.7 V (versus NHE)	C_2_H_6_ (46%)	4	0.1 m KHCO_3_ (CO_2_ sat.)	[164]
Cu 20 nma)	Thermal evaporation of Cu on GDL	H‐cell	−10.3	−0.97 V	CO (16.4%), C_2+_ (28.4%)	1	0.1 m KHCO_3_ (CO_2_ sat.)	[160]
Cu 20 nma)	PEALD of Cu on GDL	H‐cell	−31	−0.97 V	C_2_H_4_ (42%), C_2+_ (75.2%)	15	0.1 m KHCO_3_ (CO_2_ sat.)	[160]
Cu NWs	Sputtered Cu on FTO, anodized, and annealed	H‐cell	−50.9	−1.1 V	C_2_H_5_OH (14.7%), C_2_H_4_ (26.8 %), C_3_H_7_OH (3.6%)	–	0.1 m KHCO_3_ (CO_2_ sat.)	[170]
CuZn NWs	ALD of ZnO on sputtered, anodized, and annealed Cu NWs on GDL	Flow cell	−200	−0.68 V	C_2_H_5_OH (41.4%), C_2_H_4_ (18.1%), C_3_H_7_OH (5.5%)	10	1 m KOH	[170]
CuZn NPs	ALD 9:1 supercycle of Cu and Zn on GDL	H‐cell	−20	−0.90 V	CO (30.7%)	0.5	0.1 m KHCO_3_ (CO_2_ sat.)	[169]
ZnO‐Cu NPs	ALD‐ZnO of 1 cycle on ALD‐Cu on GDL	H‐cell	−20	−0.95 V	CO (20.7%)	0.5	0.1 m KHCO_3_ (CO_2_ sat.)	[169]
Cu_0.9_Zn_0.1_	Magnetron sputtering codeposition of Cu and Zn on GDL	H‐cell	−120	−1.17 V	C_2_H_4_ (38.5%), C_2_H_5_OH (32.7%)	6	1 m KOH	[110]
Au_75_Cu_25_	Magnetron sputtering codeposition of Cu and Au on Ti foil	H‐cell	−3.3	−0.7 V	CO (60%), HCOO (2%)	1	0.1 m KHCO_3_ (CO_2_ sat.)	[166]
Cu‐Au Core‐shell NWs	Magnetron sputtering of Cu on Au nanowires	H‐cell	−13	−0.65 V	CO (33%), H_2_ (67%)	24	0.5 m KHCO_3_ (CO_2_ sat.)	[192]
Ag_0.14_Cu_0.86_	Sputtering codeposition of Cu and Ag on PTFE substrate	Flow cell	−250	−0.67 V	C_2_H_5_OH (41.4%)	2	1 m KOH	[167]
Layered Ag‐Cu	Sequential sputtering of Ag and Cu (3 times) on PTFE substrate	Flow cell	−300	–	C_2_H_5_OH (26%)	–	1 m KOH	[167]
Cu_80_Ag_20_	Sputtering codeposition of Cu and Ag on GDL	Flow cell	−150	−2.62 V (versus Ag/AgCl)	C_2_H_5_OH (29.5%), C_2_H_4_ (24.3%)	1	0.5 m KHCO_3_	[168]
Cu_80_Ag_20_	Sequential sputtering of Cu and Ag on GDL	Flow cell	−150	−2.58 V (versus Ag/AgCl)	C_2_H_5_OH (23.8%), C_2_H_4_ (31.3%)	1	0.5 m KHCO_3_	[168]
Ag_20_Cu_80_	Sequential sputtering of Ag and Cu on GDL	Flow cell	−150	−2.64 V (versus Ag/AgCl)	C_2_H_5_OH (17.3%), C_2_H_4_ (24.8%)	1	0.5 m KHCO_3_	[168]
SnO_2_/Cu_6_Sn_5_/CuO	Pulsed laser sputtering of CuSn sheet on GDL	Flow cell	−70	−0.95 V	HCOOH (95.6%)	25	1 m KOH	[171]
Cu_x_Si nanostructures	CVD of butylsilane on Cu sheet	H‐cell	≈−1	−0.50 V	C_2_H_5_OH (79%)	720	0.1 m K_2_CO_3_ (CO_2_ sat.)	[173]
Cu_x_Si nanostructures	CVD of butylsilane on Cu sheet	H‐cell	≈−1	−0.65 V	CH_3_COO^−^ (72%)	720	0.1 m K_2_CO_3_ (Ar sat.)	[173]
Cu_2_Te nanosheets	CVD of Te on etched Cu foil	Flow cell	−300	–	CH_4_ (63%)	–	1.0 m KOH	[174]
CuS_x_ NPs	ALD of CuS_*x*_ on SWCNT‐O_3_ GDL with ALD‐Al_2_O_3_ adhesion layer	Flow cell	≈−5	−0.76 V	HCOO^−^ (60%)	14	0.1 m KHCO_3_	[175]
Nanowrinkled Cu	CVD of graphene on Cu sheet	H‐cell	≈ −2.5	−0.9 V	C_2_H_5_OH (40%)	6	0.1 m KCl (CO_2_ sat.)	[177]
Cu‐CNF SAC	CVD of Cu on CNFs	H‐cell	≈−10	−1.08	CO (20.6%)	–	0.1 m KHCO_3_ (CO_2_ sat.)	[172]
CuNi‐CNF DAC	CVD of Cu and Ni on CNFs	H‐cell	−20	−0.98	CO (99.6%)	25	0.1 m KHCO_3_ (CO_2_ sat.)	[172]
CeO_2_‐Cu SAC	ALD of Cu on CeO_2_	Flow cell	−400	≈ −2.5	CH_4_ (70.3%)	70	1.0 m KOH	[76]
TiO_2_‐Cu SAC	ALD of Cu on TiO_2_	Flow cell	−400	–	CH_4_ (53.7%)	–	1.0 m KOH	[76]
Al_2_O_3_‐Cu SAC	ALD of Cu on Al_2_O_3_	Flow cell	−400	–	CH_4_ (46.5%)	–	1.0 m KOH	[76]
Al_2_O_3_‐Cu NPs	ALD of Cu on Al_2_O_3_	Flow cell	−400	≈−2.0	C_2_H_4_, CH_3_COO^−^, C_2_H_5_OH (62%)	70	1.0 m KOH	[76]
Cu‐Al_2_O_3_ NCs	FS ALD of Al_2_O_3_ on Cu NCs	Flow cell	−300	−1.1 V	C_2_H_4_ (60.4 %)	24	5 m KOH	[179]

a) Film Thickness;

b) Deposition time.

Vapor deposition methods offer remarkable versatility in the synthesis of Cu‐based catalysts, including monometallic, bimetallic, and single‐atom catalysts, as well as in the modification of surface and composition of Cu substrates. Techniques such as sputtering, EB deposition, and PEALD provide precise control of morphology, allowing the tuning of surface roughness, facet orientation, and porosity to modulate local reaction environments. Moreover, optimizing the catalyst thickness via sputtering has been shown to promote C_2+_ product formation and enhance catalyst durability by stabilizing intermediate species and delaying degradation.

The key findings highlight that PVD, particularly sputtering, is the most widely used vapor deposition method due to its simplicity, scalability, and the absence of complex metal–organic precursors. However, a major limitation of PVD techniques is their tendency to produce dense, nonuniform coatings that may clog microporous structures in GDEs and limit gas diffusion. In contrast, ALD offers highly conformal and uniform coating, which is particularly beneficial for GDEs where maintaining open pathways for reactant transport is crucial. CVD, though typically requiring higher temperatures, can also produce thin compact films or high surface‐area nanostructures. This thermal requirement, however, not only increases the risk of impurity formation but may also limit the choice of substrate, particularly for GDEs that incorporate polymeric binders or carbon‐based structures. While CVD and ALD are more expensive and complex compared to PVD, their ability to deposit materials with atomic‐level control offers distinct advantages. For example, both techniques allow the synthesis of SACs and precise tailoring of metal–support interactions, which significantly enhances selectivity and stability.

Compositional tuning through cosputtering, layering, overcoating, or the use of composite precursors enables the synthesis of bimetallic and heterostructured catalysts with controlled homogeneity. These approaches introduce synergistic electronic and geometric effects that favor various products. In addition, hybrid approaches that integrate vapor deposition with other synthesis techniques provide more opportunities for advanced catalyst design. In many cases, vapor deposition techniques have been successfully employed to modify the chemical composition or the surface of the Cu substrates synthesized through methods such as electrodeposition, anodization, and colloidal techniques. Such modifications enable the incorporation of secondary elements, facet‐selective passivation, and morphological reconstruction, which fine tune the electronic structure and active site distribution for improved selectivity and catalyst stability.

Looking forward, the high growth rate and scalability of PVD can be taken advantage of to fabricate Cu electrodes, while the atomic precision of CVD and ALD can be harnessed to modify the surface or composition for tuned selectivity and higher stability. This is particularly important given the inherent instability of Cu electrodes during CO2RR, which can lead to surface reconstruction and catalyst degradation over time. Meanwhile, promising results on ALD have demonstrated its effectiveness in facet‐selective coatings, suggesting that its combination with CVD could be highly effective in improving C_2+_ selectivity. While CVD facilitates the formation of nanostructures, naturally exposing different crystal facets, ALD can then selectively deposit ultrathin oxides or modifiers, such as Al_2_O_3_ or ZnO, to fine tune catalytic behavior. Exploring this approach for Cu‐based systems could unlock new strategies for improving performance in electrochemical CO_2_ reduction.

Despite significant advances, key challenges persist in the application of vapor deposition techniques for industrially relevant electrodes. Specifically, there is a need to develop scalable and cost‐effective vapor deposition strategies that provide uniform catalyst coverage and precise structural control on GDEs. In addition, future research should also focus on integrating in situ and operando characterization with vapor deposition methods to better understand dynamic catalyst restructuring and intermediate binding under realistic reaction conditions. These insights will be critical for guiding the rational design of next‐generation Cu‐based electrocatalysts.

## Conflict of Interest

The authors declare no conflict of interest.
